# *Anastatica hierochuntica*, an *Arabidopsis* Desert Relative, Is Tolerant to Multiple Abiotic Stresses and Exhibits Species-Specific and Common Stress Tolerance Strategies with Its Halophytic Relative, *Eutrema* (*Thellungiella*) *salsugineum*

**DOI:** 10.3389/fpls.2016.01992

**Published:** 2017-01-17

**Authors:** Gil Eshel, Ruth Shaked, Yana Kazachkova, Asif Khan, Amir Eppel, Aroldo Cisneros, Tania Acuna, Yitzhak Gutterman, Noemi Tel-Zur, Shimon Rachmilevitch, Aaron Fait, Simon Barak

**Affiliations:** French Associates Institute for Biotechnology and Agriculture of Drylands, Jacob Blaustein Institutes for Desert Research, Ben-Gurion University of the NegevSde Boker, Israel

**Keywords:** abiotic stress, *Arabidopsis* relatives, *Brassicaceae*, desert plants, *Eutrema salsugineum*, extremophile plants, halophytes, extremophytes

## Abstract

The search for novel stress tolerance determinants has led to increasing interest in plants native to extreme environments – so called “extremophytes.” One successful strategy has been comparative studies between *Arabidopsis thaliana* and extremophyte *Brassicaceae* relatives such as the halophyte *Eutrema salsugineum* located in areas including cold, salty coastal regions of China. Here, we investigate stress tolerance in the desert species, *Anastatica hierochuntica* (True Rose of Jericho), a member of the poorly investigated lineage III *Brassicaceae*. We show that *A. hierochuntica* has a genome approximately 4.5-fold larger than *Arabidopsis*, divided into 22 diploid chromosomes, and demonstrate that *A. hierochuntica* exhibits tolerance to heat, low N and salt stresses that are characteristic of its habitat. Taking salt tolerance as a case study, we show that *A. hierochuntica* shares common salt tolerance mechanisms with *E. salsugineum* such as tight control of shoot Na^+^ accumulation and resilient photochemistry features. Furthermore, metabolic profiling of *E. salsugineum* and *A. hierochuntica* shoots demonstrates that the extremophytes exhibit both species-specific and common metabolic strategies to cope with salt stress including constitutive up-regulation (under control and salt stress conditions) of ascorbate and dehydroascorbate, two metabolites involved in ROS scavenging. Accordingly, *A. hierochuntica* displays tolerance to methyl viologen-induced oxidative stress suggesting that a highly active antioxidant system is essential to cope with multiple abiotic stresses. We suggest that *A. hierochuntica* presents an excellent extremophyte *Arabidopsis* relative model system for understanding plant survival in harsh desert conditions.

## Introduction

Abiotic stresses dramatically reduce crop yields leading to huge economic losses and a threat to food security (Boyer, 1982; Bray et al., 2000; Cramer et al., 2011), a problem likely to be exacerbated by climate change (Mittler and Blumwald, 2010; Lobell et al., 2011; Lesk et al., 2016). At the same time, the worldwide human population is continuously growing; from 7 billion in late 2011, it is expected to surpass 9 billion people by 2050 (United Nations, Department of Economic and Social Affairs, Population Division, 2011). Consequently, the demand for global crop production is expected to double by 2050 (Tilman et al., 2011). Yet, most new land with the potential for agriculture is situated in harsh environments such as drylands and deserts, which cover about 41% of the world land areas (Millennium Ecosystem Assessment, 2005). Therefore, the improvement of crop yields under multiple stresses is vital to prevent losses where crops are currently grown and to cultivate them on more marginal land.

The model plant, *Arabidopsis thaliana* has provided much of our understanding of the molecular responses of plants to stress. However, *Arabidopsis* is a stress-sensitive plant and thus the search for novel stress tolerance mechanisms and genes has led to increasing interest in naturally stress-tolerant plants native to extreme environments – so called “extremophytes” (John and Spangenberg, 2005; Amtmann, 2009; Dassanayake et al., 2009; Smaoui et al., 2011; Gechev et al., 2012; Oh et al., 2012; Bressan et al., 2013; Cheeseman, 2014; Flowers et al., 2015; Zhu et al., 2015). One successful strategy has been to study extremophyte *Arabidopsis* relatives (*Brassicaceae*) such as the metal hyperaccumulator *Arabidopsis halleri* (Hanikenne et al., 2008), and the halophytes, *Schrenkiella parvula* (formerly *Thellungiella parvula*) and *Eutrema salsugineum* (formerly *Thellungiella salsuginea* or *Thellungiella halophila*) (Volkov et al., 2003; Inan et al., 2004; Orsini et al., 2010; Koch and German, 2013). In addition to being salt-tolerant (e.g., Inan et al., 2004; Taji et al., 2004; Kant et al., 2006), *E. salsugineum* is also tolerant to low soil nitrogen (Kant et al., 2008), high boron levels (Lamdan et al., 2012), low phosphate levels (Velasco et al., 2016), heat stress (Higashi et al., 2013), and shows similar tolerance to cold and freezing stress as *Arabidopsis* (Griffith et al., 2007; Lee et al., 2012). A number of factors contribute to *E. salsugineum* stress tolerance including constitutive up- and down-regulation of stress tolerance genes and metabolites suggesting that *E. salsugineum* is “primed” for stress, gene copy number expansion of ion transport-related genes, sub-functionalization and neo-functionalization of duplicated genes, biased codon usage facilitating more efficient translation of proteins related to ion transportation, and the possible involvement of lineage-specific genes (Taji et al., 2004; Gong et al., 2005; Kant et al., 2006, 2008; Lugan et al., 2010; Oh et al., 2010, 2012, 2014; Sun et al., 2010; Dassanayake et al., 2011; Wu et al., 2012; Champigny et al., 2013; Kazachkova et al., 2013; Yang et al., 2013).

The various stresses to which an extremophyte exhibits tolerance reflect the combination of stresses characteristic of each specific habitat. Thus, to obtain a comprehensive understanding of how plants cope with multiple stresses, it is vital to investigate extremophytes from a range of different habitats. The Negev desert in Southern Israel, part of the Sahara and Arabian deserts belt, is characterized by a combination of temperature extremes, low water and nutrient availability, and high salinity and radiation levels, and is considered an “arid” zone with an annual rainfall of between 25 and 200 mm that is unpredictable with high spatial and temporal variation (Gutterman, 2002; Ward, 2009). Temperatures can vary both daily and seasonally between an absolute daily maximum and minimum of 46.8 and -3.6°C, respectively^1^. These extreme conditions, together with “islands of soil salinity,” have led to special morphological, physiological and genetic adaptations that allow plants to thrive. Thus, the Negev desert is a likely biogenetic treasure box of genes that are related to abiotic stress adaptations.

We have screened several of the 25 extremophyte *Brassicaceae* species that inhabit the Negev for tolerance to multiple abiotic stresses. One of these is *Anastatica hierochuntica* (True Rose of Jericho), a winter annual, Saharo-Arabian desert plant species first described by Linnaei (1753) that colonizes the uppermost, driest zones of wadies or runnels (Friedman and Stein, 1980; Friedman et al., 1981). *A. hierochuntica* is well-known due to its hygrochasy seed dispersal mechanism whereby the plant dies, dries out and reduction in turgor pressure causes the branches to curl thereby protecting the seeds and preventing their dispersal (Friedman et al., 1978; Hegazy et al., 2006). Once a serious rain event occurs causing wetting of the dead plant, the branches unfold and the seed capsules (siliculae) open. Seeds are then dispersed by raindrops hitting the open siliculae (Gutterman, 2002; Hegazy et al., 2006). Thus, although dead, *A. hierochuntica* has often been mistaken for a resurrection plant.

Here, we examine whether *A. hierochuntica* exhibits tolerance to heat, low nitrate and salt stresses associated with its desert habitat. Taking salt stress as a case study, we determine whether *A. hierochuntic*a possesses physiological salt tolerance mechanisms that are similar to its halophytic relative, *E. salsugineum*, and whether these two extremophytes from very different environments have evolved common or/and species-specific metabolic strategies for coping with salt stress.

## Materials and Methods

### Plant Material and Growth Conditions

*Anastatica hierochuntica* seeds were originally collected from the Negev Desert (455881′ N and 200751′ E), Israel. The original seed was bulked at the Ben-Gurion University experimental fields in Midreshet Ben-Gurion, and seeds from this collection were further propagated under growth room conditions (16 h light (150 μmol m^-2^ s^-1^)/8 h dark; 22°C). Seeds from the F4 generation were used for experiments presented in the current report. *A. thaliana* (Col-0), *A. hierochuntica* and *E. salsugineum* (Shandong ecotype) seeds were surface-sterilized with 50% commercial bleach for 5 min, and then rinsed four times with sterile water. *A. hierochuntica* seeds were sown onto nutrient agar plates containing MS (Murashige and Skoog, 1962) medium, pH 5.7, 0.5 g L^-1^ MES, 2% (w/v) sucrose and 0.8% (w/v) agar (Duchefa Biochemie), while *Arabidopsis* and *E. salsugineum* seeds were suspended in 0.12% agarose prior to being sown on plates. Seeds were stratified at 4°C (*Arabidopsis*, 4 days; *E. salsugineum*, 7 days; *A. hierochuntica*, 1 day) and plates incubated in the growth room [16 h light (150 μmol m^-2^ s^-1^)/8 h dark; 22°C]. Sowing of seeds from the three species was staggered so that germination occurred at approximately the same time. For soil experiments, seedlings with fully expanded cotyledons were transferred from plates to 7 cm × 7 cm × 8 cm pots containing *Arabidopsis* soil (Weizmann Institute of Science; 70% fine peat [1–10 mm], 30% perlite 4) and irrigated to field capacity with 1 g L^-1^ of 20-20-20 NPK + micronutrients solution (Haifa chemicals). Flats containing pots were covered with plastic domes and placed in the growth room. After 2 days, domes were gradually removed to allow seedling hardening. Each day, plates or pots were shuffled to remove shelf position effects. Although for the purposes of comparative studies with *Arabidopsis* and *E. salsugineum*, light conditions in the growth room were below those usually encountered by *A. hierochuntica*, **Supplementary Figure S1** shows that *A. hierochuntica* growth rates always remain lower than *Arabidopsis* even at higher light intensities. This suggests that the slower growth of *A. hierochuntica* relative to *Arabidopsis* and *E. salsugineum* was not an effect of the growth room light conditions.

### Abiotic Stress Assays

For plate-based *in vitro* analyses, heat stress (45°C) was applied for various periods and plates transferred back to the growth room for an additional 48 h. For salt stress, low NO_3_^-^ and oxidative stresses, seedlings were first sown on control MS plates. Due to differing root growth rates, *A. hierochuntica* and *Arabidopsis* seedlings were transferred to stress treatment plates, 1 and 5 days after stratification, respectively. For the root elongation assay, 10 uniform seedlings were transferred to MS vertical square plates containing various NaCl concentrations. To prevent any osmotic effects due to uptake of sucrose in the aerial tissues (MacGregor et al., 2008), the top of the agar was cut away before transfer of seedlings to ensure that shoots were not in contact with the medium. For the low NO_3_^-^ and oxidative stress assays, uniform *Arabidopsis* or *A. hierochuntica* seedlings were transferred for 5 days to vertical square plates containing MS macroelements (minus ammonium nitrate for low N experiments) and microelements, pH 5.7 (adjusted with KOH), 0.5 g L^-1^ MES, 2% (w/v) sucrose, 0.8% (w/v) agar (Duchefa Biochemie), and various concentrations of either KNO_3_^-^ or methyl viologen (MV; Sigma, M-2254).

For soil experiments, salt treatments were applied to plants with four true leaves (*Arabidopsis* and *E. salsugineum*) or two true leaves (*A. hierochuntica*). Pots were irrigated with 1 g L^-1^ of 20-20-20 NPK + micronutrients solution supplemented with various NaCl concentrations. Salt treatments commenced with 50% of the final salt concentration, and one week later, 100% percent of each salt level was applied. Plants were harvested after an additional week.

### Growth, Pigment, Ion, and Chlorophyll Fluorescence Measurements

Shoot dry weights were obtained by drying samples at 75°C for 3 days. For root elongation assays, root length was measured every 24 h for 4 days, and root relative growth rate was calculated for each consecutive day according to Hunt (1990). For oxidative stress experiments, root elongation was calculated using the formula (*L_4_-L_0_*)/*L_4_* where *L_0_* and *L_4_* are root lengths at days 1 or 4, respectively, after transfer to MV plates. For evaluating total leaf area, leaves were detached, placed between two transparency films (Graphic Vision Media), and scanned. Leaf area was calculated with ImageJ 1.43 software^2^. Anthocyanin content was analyzed according to Kant et al. (2006). Na^+^ and K^+^ ions were extracted as described by Kant et al. (2006) and ion contents determined by flame photometry (Model 410 Flame photometer, Sherwood Scientific, Ltd, UK). Chlorophyll and carotenoids were extracted in 100% methanol at a 1:10 ratio of tissue to solvent, kept overnight at 4°C in the dark, and measured according to Lichtenthaler (1987). Chlorophyll fluorescence was measured with a Mini-PAM device (Walz, Heinz Walz GmbH, Effeltrich, Germany) applying a rapid light curve (White and Critchley, 1999). Non-photochemical quenching (NPQ) and PSII ETR were calculated according to Bilger and Björkman (1990) and Krall and Edwards (1992), respectively.

### Chromosome Number and DNA Content

Chromosome number analysis was performed on young shoot tip meristem protoplasts using a drop-spread technique followed by DAPI staining and bright-field microscopy according to Anamthawat-Jonsson (2003). For flow cytometry analysis of 2C DNA content, nuclear suspensions were prepared by chopping 60–100 mg of young shoot tip tissue in 1 ml of ice-cold nuclei isolation buffer (NIB; Dolezel et al., 1989) with the addition of 5% polyvinylpirrolidone PVP-40. After addition of a further 2 ml NIB, samples were shaken for 1 h followed by filtration through 50 μm nylon mesh and centrifugation at 1,900 rpm for 8 min. The pellet containing the nuclei was resuspended in 1 ml ice-cold NIB supplemented with 50 μg m^-1^ of DNase-free RNase A and 2 μg ml^-1^ propidium iodide (Sigma-Aldrich Chemical, Co., USA), vortexed gently for a few seconds and then incubated on ice for 10 min. Samples were filtered through 35 μm mesh and run on a Becton Dickinson FACSVantage SE equipped with a 640-nm dichroic long-pass filter, a 585/42-nm band-pass filter, and an air-cooled argon-ion laser tuned to 15 mW and operating at 488 nm. For each sample, 10,000 nuclei were recorded to calculate the mean position of the G_1_ peak in the histogram. The ratio of the relative fluorescence from the G_0_/G_1_ peak positions for each sample was compared against the internal standard *Raphanus sativus* cv. Saxa as a genome size reference (2C = 1.11 pg of DNA). Cytometer gain was adjusted so that the G_0_/G_1_ peak of *R. sativus* was positioned on channel 1500.

2C DNA content was calculated as follows:

Sample 2C = (mean position of the G1 sample peak/mean position of the G1 reference peak) × reference 2C DNA.

### Metabolic Profiling

Plants were grown in soil and subjected to salt stress as described above. The fifth and sixth leaves of *Arabidopsis* and *E. salsugineum* plants, and the third and fourth leaves of *A. hierochuntica* were harvested, pooled, snap-frozen in liquid nitrogen and stored at -80°C. Four biological replicates were analyzed for each treatment and two independent experiments were carried out. Metabolite extraction was performed according to Lisec et al. (2006) with minor modifications (Kazachkova et al., 2013).

Derivatization was performed according to Lisec et al. (2006) while separation, chromatogram evaluation and metabolite identification/annotation were performed according to Kazachkova et al. (2013). Metabolite relative abundance was determined by normalizing the intensity of the peak of each metabolite to the ribitol standard, yielding ‘response’ values. These values were normalized using log_10_ transformation and analyzed statistically using MultiExperiment Viewer Version 3.1 software (Saeed et al., 2003). Two-way ANOVA tests were performed between all species and paired species. To pinpoint metabolites that were significantly affected by the salt treatment within each species (**Figure 8**), a one-way ANOVA (**Supplementary Table S3**) followed by a Dunnett test was performed. For principal component analysis (PCA), log_10_ transformed response data were used while hierarchical clustering was performed upon linear response values divided by the median abundance of each metabolite.

## Results

### *A. hierochuntica* Morphology, Genome Organization, and DNA Content

**FIGURE 1:**
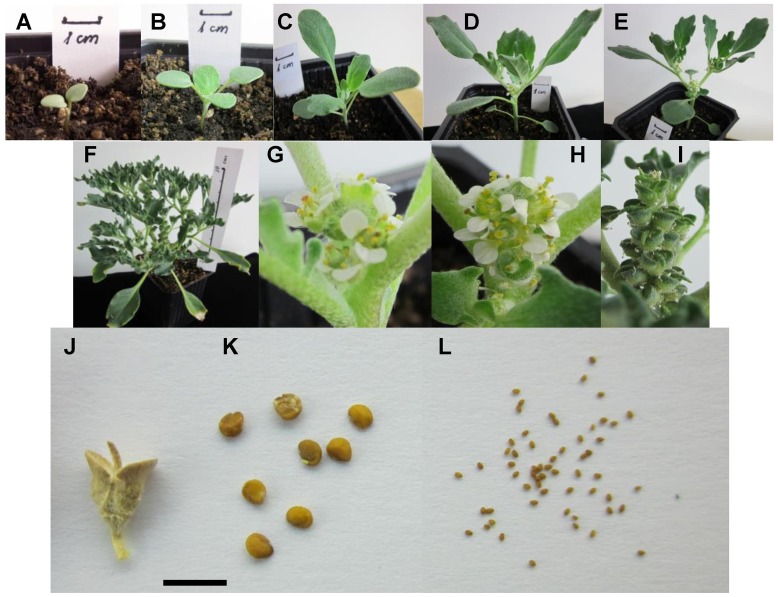
**Growth and development of *Anastatica hierochuntica*.** *A. hierochuntica* plants were raised in the growth room (see Materials and Methods) and photographed after transplantation to soil at the following times: **(A)** 4 days; **(B)** 10 days; **(C)** 19 days; **(D)** 26 days; **(E)** 31 days; **(F)** 4 months; **(G)** axillary inflorescence at branch point after 26 days; **(H)** axillary inflorescence with developing siliculae after 31 days; **(I)** siliculae on 4 month-old plant; **(J)** dry silicula; **(K)** *A. hierochuntica* seeds; **(L)** *Arabidopsis* seeds for comparison. Scale bar is 5 mm.

In the wild, *A. hierochuntica* plants exhibit great variation in developmental speed and body size ranging from between a few mm to 15–20 cm in height, depending upon the availability of soil moisture. **Figure 1** shows various stages of development of *A. hierochuntica* under our laboratory conditions. In their early stages of growth, plants are very uniform producing two cotyledons followed by two opposite pairs of obovate, densely hirsute true leaves (**Figures 1A–C**). Bifurcation of the shoot begins with the third and fourth leaves separating into individual branches. An axillary inflorescence is produced at the branch point and forms a dense cluster of sub-sessile, white flowers with four petals (**Figures 1D,G,H**). Repeated bifurcation proceeds between subsequent pairs of more lanceolate, toothed, hirsute leaves, resulting in a multi-branched, sympodial shoot structure that can vary greatly in overall three-dimensional architecture (**Figures 1E,F**). Fruits are hairy, two-winged siliculae each containing four seeds (**Figures 1I–K**).

**FIGURE 2:**
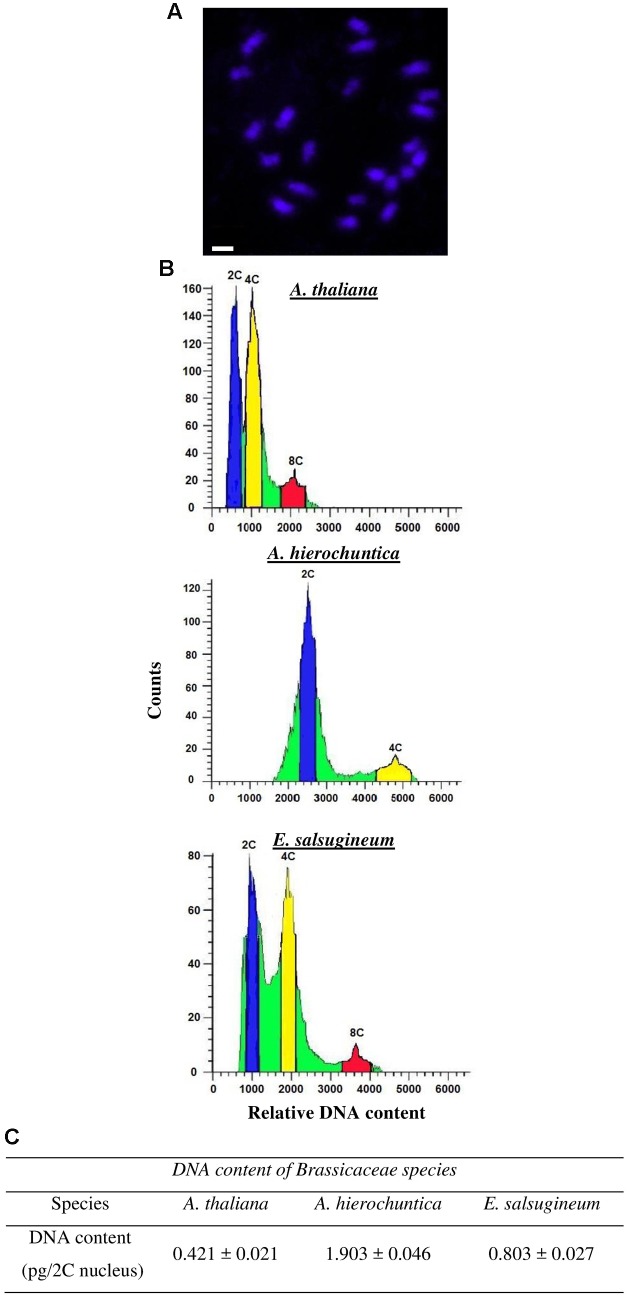
***Anastatica hierochuntica* chromosome number (diploid) and DNA content.** **(A)** DAPI-stained spread of *A. hierochuntica* chromosomes (2n = 22) in young shoot tip meristems. **(B)** Flow cytometry analysis of 2C DNA content in *Arabidopsis thaliana, A. hierochuntica* and *Eutrema salsugineum*. **(C)** DNA content of the three *Brassicaceae* species derived from **(B)**. Data are mean (*n* = 4) ± SD. The ratio of the relative fluorescence from the G_0_/G_1_ peak positions for each sample was compared against the internal standard *Raphanus sativus* cv. Saxa as a genome size reference (2C = 1.11 pg of DNA). Cytometer gain was adjusted so that the G_0_/G_1_ peak of *R. sativus* was positioned on channel 1500.

It has been reported using acid carmine staining that the *A. hierochuntica* genome is organized into 11 (haploid) chromosomes (Lifante et al., 1992). To confirm this finding, we visualized *A. hierochuntica* chromosomes using a protoplast-dropping protocol (Anamthawat-Jonsson, 2003). Analysis of 4′, 6-diamidino-2-phenylindole (DAPI) staining clearly showed the presence of 22 chromosomes (diploid) (**Figure 2A**). Flow cytometry revealed that the *A. hierochuntica* genome is approximately 4.5-fold larger than the *Arabidopsis* genome (**Figures 2B,C**). To validate our results, we also performed flow cytometry with *E. salsugineum* and showed that the genome of this species is approximately twice the size of the *Arabidopsis* genome as previously reported by Inan et al. (2004). Thus, *A. hierochuntica* has a genome size of roughly 607 Mbp.

### *A. hierochuntica* Exhibits Tolerance to Abiotic Stresses Associated with Its Desert Habitat

**FIGURE 3:**
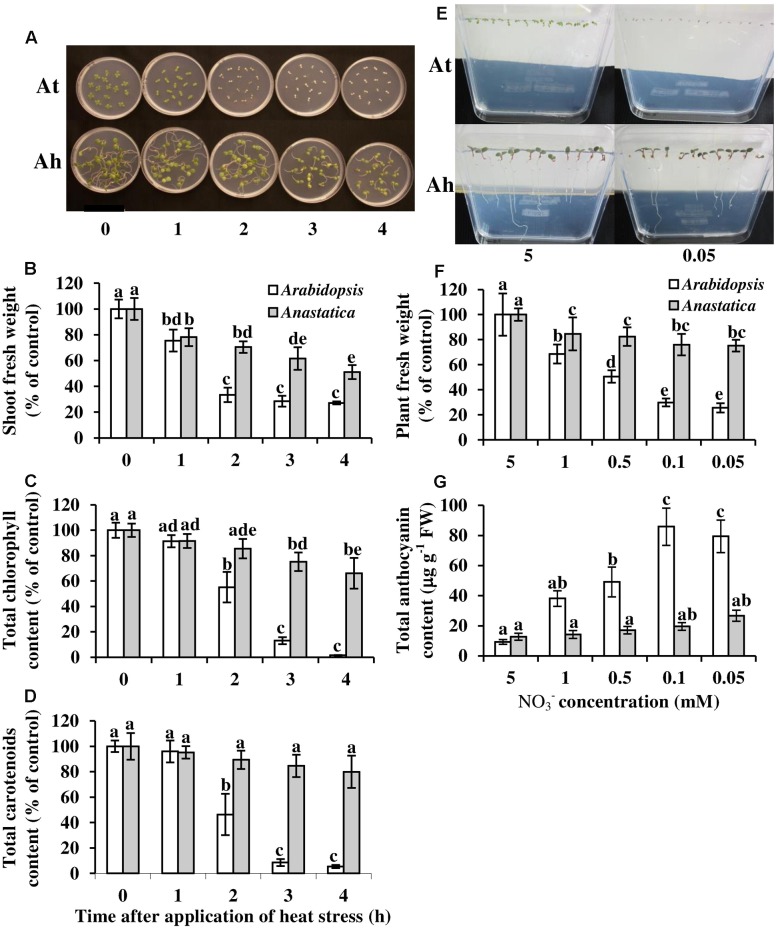
**Effect of heat shock** **(A–D)** and low KNO_3_^-^ concentrations **(E–G)** on growth parameters and pigment contents of *Arabidopsis* (At) and *A. hierochuntica* (Ah). **(A)** Picture of seedlings grown on MS medium, 48 h after exposure to 0, 1, 2, 3 or 4 h heat shock (45°C). **(B)** Shoot FW. **(C)** Total chlorophyll. **(D)** Total carotenoids. **(E)** Picture of seedlings grown for 6 days on MS medium supplemented with the indicated concentrations of KNO_3_^-^. **(F)** Plant FW. **(G)** Total anthocyanin content. Data are mean of three and four independent experiments for low NO_3_^-^ and heat, respectively ± SD. Each independent experiment comprised four replicates containing ca. 10–15 seedlings. Bars with different letters indicate a significant difference at *P* < 0.05 (Tukey HSD test). FW, fresh weight.

*Anastatica hierochuntica* plants can experience daily temperatures above 40°C. Furthermore, the Negev desert is characterized by patchy soil nitrogen pools (Zaady, 2005). For instance, we measured soil NO_3_^-^ levels ranging from 0.4 to 4 mM (data not shown). We therefore tested whether *A. hierochuntica* displays greater tolerance to heat stress and low NO_3_^-^ levels in the growth medium, than *Arabidopsis*. *In vitro*-grown seedlings (two-cotyledon stage) of both species survived 1 h of heat stress (45°C) with an approximate 25% reduction in fresh weight (FW) for both species but no effect on chlorophyll or carotenoid content (**Figures 3A–D**; **Supplementary Figure S2**). Two hours of heat stress severely affected *Arabidopsis* seedlings leaving them pale or bleached (**Figure 3A**). This effect was reflected in a 67% reduction in FW, and a 45 and 54% reduction in chlorophyll and carotenoid content, respectively, compared to control seedlings. On the other hand, there was no further reduction in the FW of *A. hierochuntica* seedlings compared to 1 h heat stress and no effect on chlorophyll and carotenoid levels compared to the control. Heat stress of 3 and 4 h caused severe stress to *Arabidopsis* seedlings as manifest by no further growth of seedlings and almost complete bleaching of cotyledons. In contrast, *A. hierochuntica* displayed considerably greater heat tolerance than *Arabidopsis* after 3 and 4 h of heat stress. For instance, 4 h heat stress, caused a 50% drop in *A. hierochuntica* FW whereas *Arabidopsis* exhibited a 73% FW reduction. Furthermore, *A. hierochuntica* seedlings did not become bleached but maintained chlorophyll levels at approximately 65% of control levels with no effect of heat stress on carotenoid content.

*Anastatica hierochuntica* also displayed tolerance to low N stress compared to *Arabidopsis* (**Figure 3E**). *Arabidopsis* showed a progressive, sharp reduction in FW as NO_3_^-^ levels were reduced in nutrient agar, exhibiting a 75% reduction in FW at 0.05 mM NO_3_^-^ (**Figure 3F**; **Supplementary Figure S3**). In contrast, *A. hierochuntica* showed a much more moderate drop in FW as NO_3_^-^ levels were reduced, and FW only fell by 25% at 0.05 mM NO_3_^-^. The highly stressed condition of the *Arabidopsis* seedlings at low NO_3_^-^ levels was manifest by the production of progressively higher amounts of anthocyanins as NO_3_^-^ levels dropped (**Figure 3G**). On the other hand, no significant differences in *A. hierochuntica* anthocyanin content compared to control could be discerned at any NO_3_^-^ level.

**FIGURE 4:**
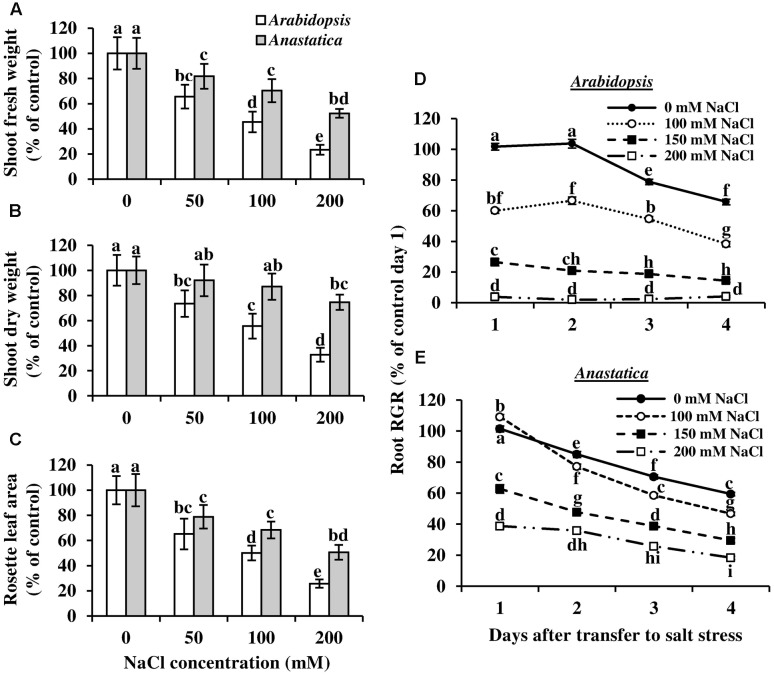
**The effect of salt stress on growth parameters of *A. hierochuntica* and *Arabidopsis*.** **(A–C)** Plants grown on soil were exposed to incremental increases of NaCl concentration, and were harvested 1 week after the final NaCl concentration was reached. **(A)** Shoot FW. **(B)** Shoot DW. **(C)** Rosette leaf area. **(D,E)** Seedlings were grown on MS medium in vertical plates without NaCl and then transferred to fresh vertical MS plates supplemented with the indicated NaCl concentrations. **(D)** *Arabidopsis* root relative growth rate (RGR). **(E)** *A. hierochuntica* root RGR. Data are expressed as a percentage of control (0 mM) RGR on day 1 after transfer to salt stress. Data are mean of three to four experiments ± SD. Each independent experiment comprised four replicate plates with 10 seedlings per plate. For both soil and plate experiments, bars or time points with different letters indicate a significant difference at *P* < 0.05 (Tukey HSD test). FW, fresh weight; DW, dry weight.

Because the Negev desert possesses islands of high salinity and *A. hierochuntica* is also found growing in the Dead Sea valley, we tested whether *A. hierochuntica* exhibits greater tolerance to salt stress than *Arabidopsis*. A much greater reduction in *Arabidopsis* growth was discerned as salt concentration increased than was observed in *A. hierochuntica* (**Figures 4A–C**; **Supplementary Figure S4**). For instance, at 100 and 200 mM NaCl, *Arabidopsis* displayed a 55 and 77% reduction in FW, respectively, while *A. hierochuntica* FW was only reduced by 30 and 48% at the respective iso-saline concentration (**Figure 4A**). Similarly, dry weight was affected to a lesser extent in *A. hierochuntica* than *Arabidopsis* at 100 and 200 mM NaCl (**Figure 4B**). *Arabidopsis* leaf area was also affected to a greater extent by salt stress than *A. hierochuntica*. For example, *Arabidopsis* exhibited a 74% reduction in leaf area at 200 mM NaCl whereas *A. hierochuntica* leaf area only dropped by 49% (**Figure 4C**). Furthermore, in an *in vitro* plate root elongation assay, *Arabidopsis* seedlings showed a clear and drastic salt-mediated dose-response reduction in root relative growth rate such that at 200 mM NaCl, root elongation had virtually ceased (**Figure 4D**). On the other hand, 100 mM NaCl only had a mild effect on *A. hierochuntica* root relative growth rate while root elongation continued even at 200 mM NaCl (**Figure 4E**).

### *A. hierochuntica* Tightly Controls Na^+^ Accumulation and Exhibits Photochemical Characteristics that Are Resilient to Salt Stress

**FIGURE 5:**
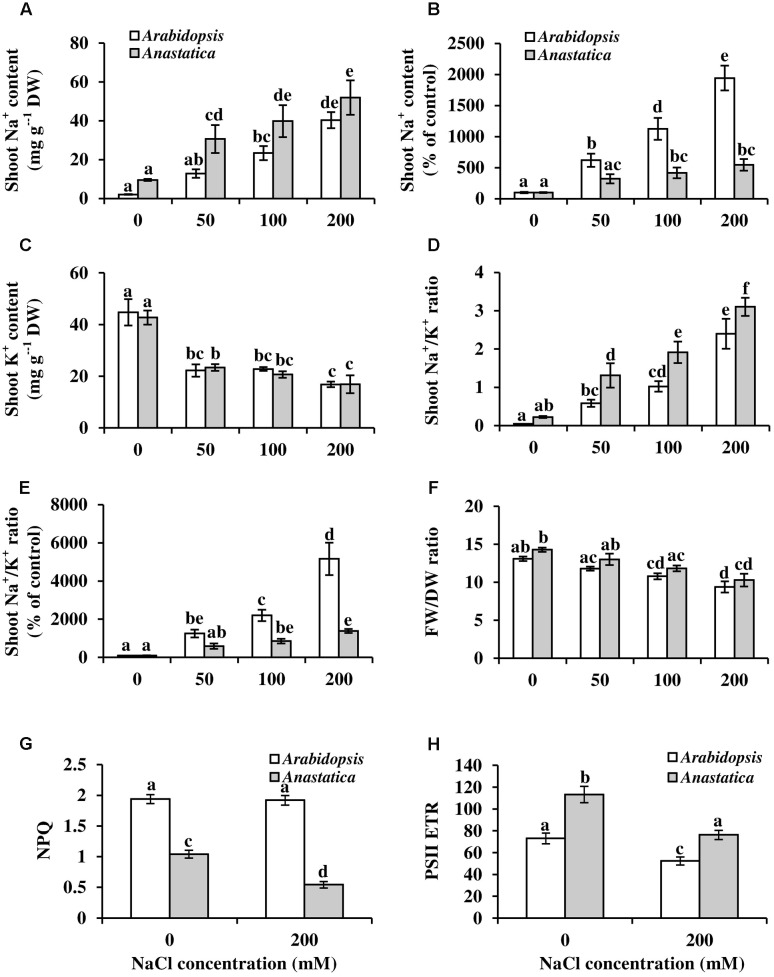
**Effect of NaCl on Na^+^ and K^+^ contents** **(A–D)** and photochemistry parameters **(E,F)** of *Arabidopsis* and *A. hierochuntica* shoots. Plants grown on soil were exposed to incremental increases of NaCl concentration. The plants were harvested 1 week after the final NaCl concentration was reached. **(A)** Absolute Na^+^ content. **(B)** Relative changes in Na^+^ content compared to control treatment. **(C)** K^+^ content. **(D)** Na^+^/K^+^ ratio. **(E)** Relative changes in Na^+^/K^+^ ratio compared to control treatment. **(F)** Shoot FW/DW ratio. **(G)** Non-photochemical quenching (NPQ). **(H)** PSII electron transport rates (ETR). Values of NPQ and PSII ETR are presented from plants exposed to a light intensity of 1500 Photosynthetic Photon Flux Density (PPFD). Data represent mean (*n* = 4) ± SD. Each replicate comprised pooled data from six individual plants. The data are representative of similar results from two independent experiments. Bars with different letters indicate a significant difference at *P* < 0.05 (Tukey HSD test). FW, fresh weight; DW, dry weight.

One feature of natural salt tolerance is the ability of plants to tightly control Na^+^ accumulation (e.g., Kant et al., 2006). Under control conditions, *A. hierochuntica* possessed about 4.5 times as much Na^+^ in the shoots as *Arabidopsis*, similar to the threefold higher amount of *E. salsugineum* shoot Na^+^ compared to *Arabidopsis* (**Figure 5A**; Kant et al., 2006). Both species accumulated increasing amounts of Na^+^ as salt concentrations increased, but *Arabidopsis* accumulated Na^+^ to a much greater extent than *A. hierochuntica* (**Figure 5B**). *Arabidopsis* and *A. hierochuntica* exhibited comparable shoot K^+^ levels under control conditions and a similar reduction in K^+^ content in response to salt stress (**Figure 5C**). Thus, under control conditions, *A. hierochuntica* had a higher Na^+^/K^+^ ratio than *Arabidopsis* (**Figure 5D**) but as soil salt concentrations increased, Na^+^/K^+^ ratios rose to a much lower extent in *A. hierochuntica* (**Figures 5D,E**). Both species exhibited similar FW/DW ratios at all salt concentrations (**Figure 5F**) suggesting that differences in ion contents was not due to variations in tissue water content. Taken together, our ion analysis data suggest that *A. hierochuntica* is able to tightly control Na^+^ accumulation.

We recently reported that *A. hierochuntica* under control conditions exhibits lower NPQ, a mechanism for dissipating excess light energy, than *Arabidopsis*, and a relatively higher photosystem II electron transport rate (PSII ETR) (Eppel et al., 2014). In the present study, *A. hierochuntica* exhibited an approximately 50% lower NPQ than *Arabidopsis* under control conditions (**Figure 5G**), and while salt stress had no effect on *Arabidopsis* NPQ, it caused a further 50% fall in *A. hierochuntica* NPQ. Under control conditions, PSII ETR was about 55% higher in *A. hierochuntica* compared to *Arabidopsis* (**Figure 5H**). Salt stress caused a fall in PSII ETR in both species but *A. hierochuntica* still maintained a 45% higher PSII ETR in comparison to *Arabidopsis*. Thus, the NPQ and PSII ETR data suggest that *A. hierochuntica* photochemistry displays considerable resilience to salt stress.

### *Arabidopsis*, *E. salsugineum*, and *A. hierochuntica* Metabolic Reprogramming in Response to Salt Stress

*Eutrema salsugineum* exhibits extreme salt tolerance (Kant et al., 2006; Kazachkova et al., 2013), which presented us with the opportunity of comparing three related species originating from very different environments, and differing in their salt tolerance: the salt-sensitive glycophyte *Arabidopsis* (temperate Columbia, MT, USA), and the two extremophytes – moderately salt-tolerant *A. hierochuntica* (hot, dry Negev desert), and highly tolerant *E. salsugineum* (cold, salty coastal areas of the Shandong province, China).

**FIGURE 6:**
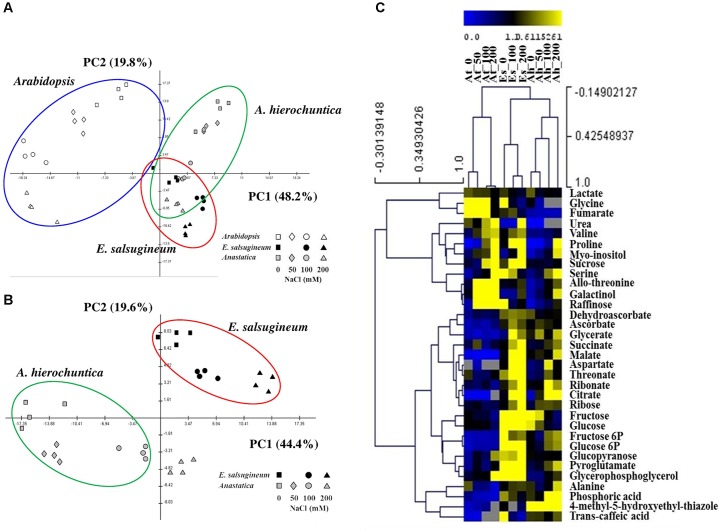
**Principal component analyses (PCA) and hierarchical clustering of metabolic profiles of *Arabidopsis* (At), *A. hierochuntica* (Ah) and *E. salsugineum* (Es) under control and salt stress conditions.** For PCA, metabolite relative abundance was first normalized using log_10_ transformation and then analyzed statistically using MultiExperiment Viewer Version 3.1 software (Saeed et al., 2003). The variance explained by each component is indicated in brackets. **(A)** PCA comparing all three species. **(B)** PCA comparing *E. salsugineum* and *A. hierochuntica*. **(C)** Hierarchical clustering (HCA) of all three species. For HCA, averages of absolute values (*n* = 4) for each metabolite normalized by the ribitol standard were divided by the median normalized metabolite abundance and are presented as a heat map. For both analyses, data are representative of similar results from two independent experiments.

*Arabidopsis*, *E. salsugineum*, and *A. hierochuntica* plants grown in soil were exposed to increasing concentrations of NaCl (*E. salsugineum* was not exposed to 50 mM NaCl due to the mild effect of this concentration; Kazachkova et al., 2013), and the relative abundance of primary carbon and nitrogen metabolites was determined for leaf tissue. To gain a global view of the differences between the plant species and across treatments, PCA was employed. Inspection of the first two principle components, which account for 68% of the total variance within the data set, allowed classification of samples by species and treatments (**Figure 6**). The first principal component accounting for 48.2% of total variance clearly separated the metabolism of *Arabidopsis* from its two extremophyte relatives (**Figure 6A**). Among the metabolites most affecting the separation between species (in decreasing order of absolute eigenvector value and confirmed by two-way ANOVA) were raffinose, malate, galactinol, fumarate, phosphoric acid, and glycerate (**Supplementary Tables S1** and **S2**). Overall, the two-way ANOVA test for all three species revealed that 25 out of 33 metabolites were significantly different between the species (**Supplementary Table S2**). Among these 25 metabolites, 22 metabolites also displayed a significant species × salt-treatment interaction.

The second principal component accounting for 19.8% of total variance discriminated samples according to salt treatment. Among the metabolites most affecting separation between samples (in descending order of absolute eigenvector value and confirmed by two-way ANOVA) were proline, malate, raffinose, serine, fumarate, and myo-inositol. Overall, 20 metabolites were found to be significantly different between salt treatments (**Supplementary Table S2**). Control and salt-treated *Arabidopsis* sample groups were substantially spread out across the second component, whereas *E. salsuginem* and *A. hierochuntica* sample groups were more condensed.

The complete separation of the metabolic profiles of *Arabidopsis* and the two extremophytes, and the partially overlapping metabolic response of *E. salsugineum* and *A. hierochuntica* suggested both common and species-specific responses of the extremophytes to salt stress (**Figure 6A**). This notion was supported by hierarchical clustering analysis (HCA) that placed the two extremophytes on a separate clade from *Arabidopsis* but separated *E. salsugineum* and *A. hierochuntica* on two sub-clades (**Figure 6C**). PCA of the two extremophytes alone clearly illustrated the species-specific metabolic response to salt stress (**Figure 6B**). The metabolites that primarily affected the separation of the extremophytes (in decreasing order of absolute eigenvector value and confirmed by two-way ANOVA test) were: proline, citrate, aspartate, raffinose, threonic acid, and pyroglutamic acid (**Supplementary Tables S1** and **S2**).

**FIGURE 7:**
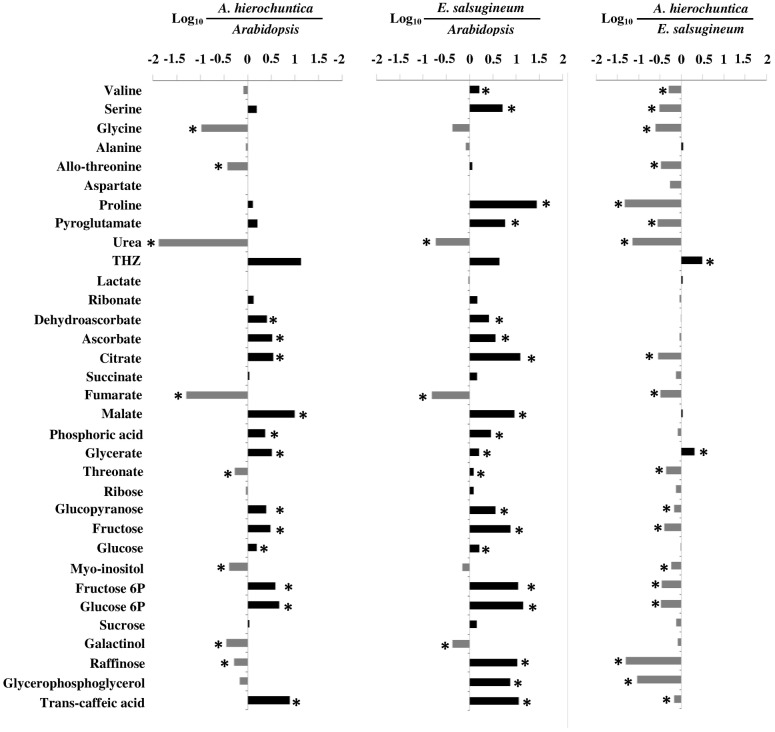
**Comparison of metabolites between *Arabidopsis*, *E. salsugineum*, and *A. hierochuntica* under control conditions.** Data are representative of similar results from two independent experiments. Metabolites that significantly differ between species are marked with asterisks (Student’s *t*-test, *P* < 0.05, *n* = 4). Black and gray bars indicate metabolites that are more abundant in the numerators or denominators, respectively. THZ, 4-methyl-5-hydroxyethyl-thiazole.

Examination of individual metabolites under control conditions revealed an overall comparable metabolic phenotype between the two extremophytes with most of the metabolites exhibiting a significantly higher abundance in *A. hierochuntica* and *E. salsugineum* compared to *Arabidopsis* (**Figure 7**). Among the metabolites exhibiting differences between *Arabidopsis* and the two extremophytes under control conditions were: (i) the TCA cycle intermediates, citrate and malate that were significantly higher in the extremophytes, and fumarate which was significantly higher in *Arabidopsis*; (ii) the sugars, glucose and fructose; (iii) the hexose phosphates, fructose-6-phosphate and glucose-6-phosphate; (iv) phosphoric acid; (v) the antioxidants, ascorbate and dehydroascorbate (Foyer and Noctor, 2011). The levels of the metabolites indicated in (ii) to (v) were all higher in the extremophytes. In addition, the osmoprotectant proline (Smirnoff and Cumbes, 1989) was higher in the extremophyte plants under control conditions while the osmoprotectants myo-inositol (also a precursor of the osmoprotectant galactinol; Bohnert et al., 2006) and galactinol (also a precursor of the osmoprotectant raffinose; Taji et al., 2002; Nishizawa et al., 2008) were higher in *Arabidopsis*. Interestingly, the majority of metabolites displayed significantly lower levels in *A. hierochuntica* compared to *E. salsugineum*.

**FIGURE 8:**
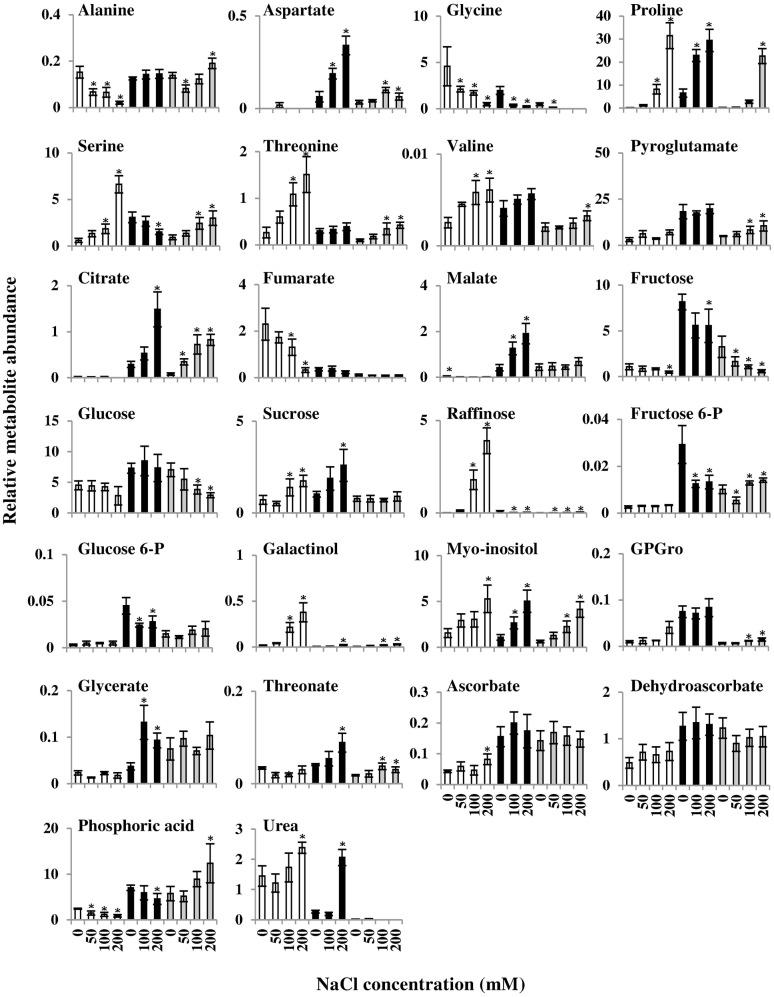
**Comparison of *Arabidopsis* (white bars), *E. salsugineum* (black bars), and *A. hierochuntica* (gray bars) metabolic responses to salt stress.** Data are mean (*n* = 4) ± SD, and are representative of two independent experiments. Asterisks represent significant difference (*P* < 0.05, Dunnett test) between *Arabidopsis*, *E. salsugineum* or *A. hierochuntica* and their respective controls. GPGro, glycerophosphoglycerol.

Inspection of the levels of metabolites in response to salt stress revealed only a few common metabolite responses. All three species exhibited increased levels of the osmoprotectants myo-inositol and proline in response to salt stress (**Figure 8**). Notably, *E. salsugineum* exhibited greater proline content than *Arabidopsis* and *A. hierochuntica* at control and 100 mM NaCl. High proline levels are a well-characterized feature of soil-grown, well-fertilized *E. salsugineum* (Kant et al., 2006; Guevara et al., 2012; Kazachkova et al., 2013). In addition, glycine levels were reduced in response to stress in all three species although the actual level of glycine was highest in *Arabidopsis* at all treatment levels compared to the extremophytes.

In contrast to the few common metabolic responses between the three species, a considerable number of metabolites exhibited an overall common response in both extremophytes that was different to that observed in *Arabidopsis*. For instance, among amino acids, the level of alanine in *Arabidopsis* gradually declined in response to salt, while remaining constitutively high in the extremophytes and even rising slightly in *A. hierochuntica* at 200 mM NaCl. Aspartate was barely detectable in *Arabidopsis*, yet increased substantially with salt concentration in both extremophyte species, but particularly in *E. salsugineum*. In contrast, the levels of threonine increased dramatically in *Arabidopsis*, while remaining relatively lower in the extremophytes, with a small but significant rise in *A. hierochuntica* at higher salt levels. Serine and glycerate, which are intermediates of the photorespiratory cycle, also displayed differences in accumulation between the extremophytes and *Arabidopsis*. Serine content increased in response to salt in both *Arabidopsis* and *A. hierochuntica* but accumulated to considerably higher levels in *Arabidopsis* at the highest salt concentration. On the other hand, serine only displayed a slight salt-mediated decrease in *E. salsugineum*. Overall glycerate levels were much lower in *Arabidopsis* than in the extremophytes. Furthermore, while glycerate exhibited no response to salt in *Arabidopsis*, it was maintained at a constantly higher level in *A. hierochuntica* and displayed a salt-mediated increase *in E. salsugineum*.

The levels of the TCA cycle intermediates, citrate and malate, were substantially higher in the extremophytes compared to *Arabidopsis*. However, whereas in *E. salsugineum* both citrate and malate levels increased significantly in response to salt, citrate levels in *A. hierochuntica* increased significantly while malate remained at a constantly higher level than in *Arabidopsis*. Fumarate content, on the other hand, was substantially higher in *Arabidopsis*, decreasing in response to salt stress, but remaining constantly low in the extremophytes. The levels of other carboxylic acids, such as ascorbate and dehydroascorbate, were constitutively higher in the extremophytes than in *Arabidopsis*.

Differences in sugar contents between *Arabidopsis* and the extremophytes were also observed. The most striking differences were in the levels of the osmoprotectants galactinol and raffinose (Taji et al., 2002; Nishizawa et al., 2008). Both increased dramatically in *Arabidopsis*, but were induced to a much lower extent in the extremophytes. In addition, fructose-6-phosphate and glucose-6-phosphate accumulated to a greater extent in the extremophytes, being maintained at overall constant levels in *A. hierochuntica*, while significantly reduced in *E. salsugineum* in response to salt.

We also discerned species-specific responses that differentiated between the extremophytes. For example, the level of serine in *E. salsugineum* was significantly reduced by salt stress whereas in *A. hierochuntica* serine content increased significantly. Pyroglutamate (possibly part of the glutathione recycling pathway, an osmoprotectant and a Glu reservoir (Ohkama-Ohtsu et al., 2008; Kumar and Bachhawat, 2012; Schreiber et al., 2012), fructose, and glycerophosphoglycerol all exhibited constitutively higher levels in *E. salsugineum* compared to *A. hierochuntica*.

### *A. hierochuntica* is Tolerant to Severe Oxidative Stress

**FIGURE 9:**
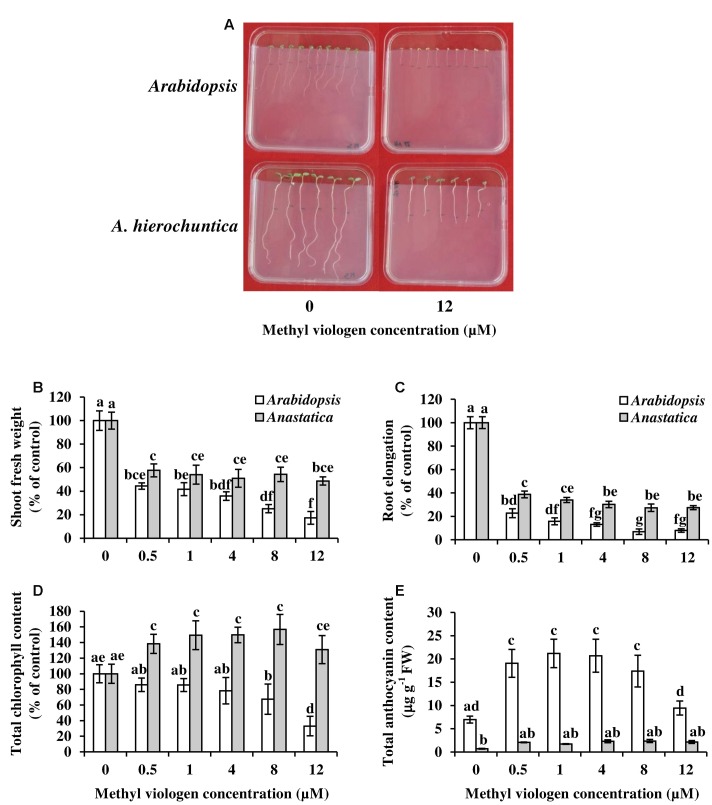
**The effect of oxidative stress on growth parameters and pigment contents of *Arabidopsis* and *A. hierochuntica*.** Seedlings grown on vertical MS plates were transferred to 0, 0.5, 1, 4, 8, or 12 μM Methyl viologen for 5 days. **(A)** Phenotypes of seedlings after 5 days on 0 and 12 μM Methyl viologen. **(B)** Shoot FW. **(C)** Root elongation. **(D)** Total chlorophyll content. **(E)** Total anthocyanin content. Data are mean (*n* = 4) ± SD. Each replicate plate contained ca. 10 (*Arabidopsis*) or ca. 6 (*A. hierochuntica*) seedlings. Data are representative of two independent experiments. Bars with different letters indicate a significant difference at *P* < 0.05 (Tukey HSD test). FW, fresh weight.

The constitutively higher levels of the antioxidant compounds ascorbate and dehydroascorbate in *A. hierochuntica* (and *E. salsugineum*) compared to *Arabidopsis* (**Figures 7** and **8**) suggested that *A. hierochuntica* possesses a constitutively active antioxidant system. Comparison of the response of *Arabidopsis* and *A. hierochuntica* seedlings to ROS-forming methyl viologen (MV) (Babbs et al., 1989; Fujii et al., 1990), demonstrated that *A. hierochuntica* seedlings exhibited remarkable tolerance to increasing concentrations of MV compared to *Arabidopsis* (**Figure 9A**). *A. hierochuntica* shoot FW and root elongation decreased to a far lesser extent than *Arabidopsis* as MV concentration rose. Furthermore, *A. hierochuntica* maintained total chlorophyll levels in response to extreme MV concentrations (12 μM) whereas chlorophyll levels were reduced in *Arabidopsis*, which exhibited signs of bleaching (**Figures 9A–D**; **Supplementary Figure S5**). The severe stress symptoms observed in *Arabidopsis* were also reflected in the high accumulation of anthocyanins in response to MV whereas only low levels of anthocyanins were observed in *A. hierochuntica* (**Figure 9E**). These data suggest that *A. hierochuntica* has a highly active antioxidant system.

## Discussion

### *Anastatica hierochuntica* – A Lineage III Desert Plant that is Tolerant to Multiple Abiotic Stresses

The *Brassicaceae* family comprises over 3,600 species including many important crops as well as the premier model plant system *A. thaliana* (Franzke et al., 2011; Al-Shehbaz, 2012). It contains 301 genera classified into 49 monophyletic tribes. The family can be split into two major groups: the *Aethionema* group and the core group (Franzke et al., 2011), the latter of which can be divided into three major lineages (lineages I, II, and III).

In recent years, a number of genomic resources have been generated for the *Brassicaceae* thereby providing an impressive platform for evolutionary and comparative biology studies. At least 36 genomes have been sequenced or are being sequenced (Koenig and Weigel, 2015) including 13 species from lineage I (e.g., *A. thaliana*, *Arabidopsis lyrata*, *Capsella rubella*), 17 species from lineage II or extended lineage II (e.g., *Arabis alpina*, *Brassica napus*, *E. salsugineum*, *S. parvula*) but only three species from lineage III (*Diptychocarpus strictus*, *Euclidium syriacum, Malcolmia maritima*) (Franzke et al., 2011; Kiefer et al., 2014; Huang et al., 2015). Moreover, of the completed genomes, none are from lineage III species. This rich collection of genomes affords a means of understanding characteristics such as developmental and life history traits that may be missing in *A. thaliana*. In particular, extremophytes of the *Brassicaceae* family that thrive in some of the most extreme environments offer excellent systems for comprehending the molecular mechanisms underlying adaptation to harsh environments.

The extremophyte *A. hierochuntica* from the tribe *Anastaticeae* belongs to the underrepresented lineage III species (Couvreur et al., 2010; Kiefer et al., 2014; Huang et al., 2015), for which there is a dearth of physiological and molecular knowledge. Moreover, *A. hierochuntica* presents an opportunity for comparison of a desert annual with *Brassicaceae* extremophytes such as *E. salsugineum* that will facilitate our understanding of how plants have adapted to very different extreme habitats.

In the current study, we demonstrated that *A. hierochuntica* is tolerant to several stresses associated with its desert habitat, namely high temperatures, low soil N, and salinity (**Figures 3** and **4**). Both heat tolerance and low N tolerance are important traits for which *A. hierochuntica* might be a source of novel tolerance determinants. Heat stress, especially when combined with drought, can lead to huge losses in crop production (Mittler, 2006; Mittler and Blumwald, 2010; Lesk et al., 2016) particularly because cereals are most sensitive to these stresses during the grain-filling phase (Barnabas et al., 2008). Indeed, a recent report identified heat tolerance in wheat at the reproductive stage as being a key trait for increasing yields under projected climate change (Stratonovitch and Semenov, 2015). Although detailed reports on extremophytes able to withstand extremely high temperatures are sparse, some work has begun (Emad El-Deen, 2005; Lawson et al., 2014; Yates et al., 2014; Gallas and Waters, 2015). *Rhazya stricta* in particular, can maintain fully functional photosynthesis in the field at leaf temperatures as high as 43°C along with light intensities >1000 μmol photons m^-1^ s^-1^ (Lawson et al., 2014).

Nitrogen use efficiency (NUE) is another trait which has been the focus of much research and breeding efforts (McAllister et al., 2012; Han et al., 2015). Crop plants only exploit about 30 to 40% of applied N (Raun and Johnson, 1999), and the remaining N is lost by leaching, denitrification, volatilization, soil erosion, and microbial consumption thereby increasing both environmental N pollution and production costs (Good et al., 2004). Because traditional breeding for improved NUE has hit a plateau, the use of low N-tolerant extremophytes has been suggested as one approach to identify new alleles and genes (Kant et al., 2011). Indeed, *E. salsugineum* is more tolerant to N-limiting conditions than *Arabidopsis*, and possesses higher N content, total amino acids and total soluble protein at low soil N levels (Kant et al., 2008). The higher NUE of *E. salsugineum* can be partly attributed to constitutively higher NO_3_^-^ uptake under low N conditions, as well as differential expression of genes encoding NO_3_^-^ assimilation and transporter genes.

### *A. hierochuntica* and *E. salsugineum* Share Similar Salt Uptake and Photosynthetic Characteristics in Response to Salt Stress

*Anastatica hierochuntica* exhibited several physiological features of salt tolerance that are similar to those observed in *E. salsugineum* (Inan et al., 2004; Kant et al., 2006). Firstly, under control conditions, *A. hierochuntica* possesses higher shoot Na^+^ levels than *Arabidopsis* (**Figure 5**). In *E. salsugineum*, higher shoot Na^+^ content probably aids in maintaining a constitutively more negative leaf osmotic potential than *Arabidopsis* (Inan et al., 2004). Secondly, under saline conditions *A. hierochuntica* exhibits tight control of Na^+^ accumulation compared to *Arabidopsis* (**Figures 5A–D**) again similar to *E. salsugineum* (Kant et al., 2006). A significantly reduced Na^+^ inward current and smaller Na^+^ influx into *E. salsugineum* roots compared to *Arabidopsis* (Volkov and Amtmann, 2006; Wang et al., 2006), as well as differential expression of a gene encoding the Na^+^/H^+^ antiporter, SOS1 (Kant et al., 2006), likely contribute to stringent control of Na^+^ uptake in the extremophyte.

Thirdly, *A. hierochuntica* displays a higher PSII electron transport and a lower NPQ than *Arabidopsis* under control and saline conditions suggesting that a greater amount of light energy is used for productive purposes in *A. hierochuntica* than in *Arabidopsis* (**Figures 5G,H**). *E. salsugineum* is also able to maintain a lower NPQ than *Arabidopsis* under saline conditions while PSII electron transport actually increases in response to salt (Stepien and Johnson, 2009).

### *A. hierochuntica* Possesses a Highly Active Antioxidant System

*Anastatica hierochuntica* displays constitutively high levels of antioxidant compounds (**Figures 7** and **8**) and striking tolerance to ROS-generating MV (**Figure 9**) suggesting that *A. hierochuntica* possesses a highly active antioxidant system, which is a common feature of extremophytes. In *E. salsugineum* and *S. parvula* for example, several enzymes involved in ROS scavenging are highly active, and antioxidant metabolites such as thioredoxin, ascorbate, and dehydroascorbate exhibit constitutively higher levels than in *Arabidopsis* (M’rah et al., 2007; Kazachkova et al., 2013; Uzilday et al., 2014). Higher expression of *E. salsugineum* genes encoding components of the antioxidant machinery under both control and salt stress conditions might underlie the higher enzyme activities and antioxidant levels (Taji et al., 2004; Gong et al., 2005).

Active antioxidant mechanisms are not only important for desert extremophytes or halophytes but are also crucial for other extremophytes. For instance, arctic plants such as *Deschampsia antarctica*, have to sustain photochemistry under a combination of low temperatures and high light. *D. antarctica* possesses constitutively higher superoxide dismutase (SOD) activity compared to other *Poaceae*, expresses a hydrogen peroxide-resistant MnSOD isozyme during cold acclimation, and produces a number of inducible UV-B protective compounds (Xiong and Day, 2001; Perez-Torres et al., 2004; Ruhland et al., 2005).

In addition to antioxidant mechanisms, energy dissipating processes can also prevent generation of damaging ROS. For instance, in extremophytes such as *A. hierochuntica* and the alpine plant, *Ranunculus glacialis*, photorespiration plays a role in energy dissipation (Streb et al., 2005; Eppel et al., 2014). However, this does not appear to be the case in *E. salsugineum*. Rather, there is a stress-mediated rise in the protein levels of plastid terminal oxidase that could function as an alternative electron sink and prevent generation of ROS (Stepien and Johnson, 2009). Interestingly, the amount of PTOX in *R. glacialis* leaves exceeds the amount found in all other plants species examined, and even when electron flow to assimilation, photorespiration and the Mehler reaction are blocked, *R. glacialis* leaves under high light intensity can maintain electron flow (Streb et al., 2005; Laureau et al., 2013).

Thus, for extremophytes encountering a combination of severe stresses such as *A. hierochuntica* (high light intensity, high temperatures, low soil nutrient status, saline soils), highly active antioxidant systems often coupled with excess energy dissipation mechanisms appear crucial for survival in extreme habitats.

### Extremophytes from Different Habitats Exhibit Common and Distinct Metabolic Strategies for Coping with Salt Stress

Our metabolic profiling data suggested that the extremophytes possess both common and species-specific stress-mediated changes in metabolism that are different from *Arabidopsis* (**Figures 6–8**). Furthermore, the PCA analysis suggested that the different levels of salt stress cause much larger changes in *Arabidopsis* than in the extremophytes (**Figure 6**) indicating that *A. hierochuntica* and *E. salsugineum* may be primed for stress in agreement with other reports on *E. salsugineum* (e.g., Kant et al., 2006; Kazachkova et al., 2013; Vera-Estrella et al., 2014). Further support for the notion that the extremophytes are primed for stress could be seen in the analysis of metabolite levels under control conditions (**Figure 7**). Both extremophytes showed accumulation of antioxidants, sugars and TCA cycle intermediates, which have been previously shown to be important for plant responses to various stresses, including salt and drought (Smirnoff, 1996; Urano et al., 2009; Lugan et al., 2010; Obata and Fernie, 2012). Not all extremophytes, however, are primed for stress. For example, the salt tolerant extremophyte legume *Lotus creticus* exhibits no such phenomenon (Sanchez et al., 2011).

Striking differences were observed between the extremophytes and *Arabidopsis* in the abundance of specific TCA cycle intermediates. Both extremophytes exhibited high levels of malate and citrate but low levels of fumarate whereas *Arabidopsis* showed the opposite pattern (**Figures 7** and **8**). This metabolic signature appears to be an important feature of extremophyte *Brassicaceae* under control, salt and low-N conditions and irrespective of growth platforms (Kant et al., 2008; Kazachkova et al., 2013). We previously speculated that a large increase in the malate pool and low fumarate levels could reflect the generation of oxaloacetate to provide carbon skeletons for enhanced amino acid synthesis in *E salsugineum* (Kazachkova et al., 2013). Oxaloacetate can be further metabolized to aspartate (Sweetlove et al., 2010), and indeed we observed higher control and salt-induced levels of aspartate in the extremophytes compared to *Arabidopsis*. One use of higher aspartate levels might be to supply amino groups to the photorespiratory cycle via glycine (Novitskaya et al., 2002). Alanine can also contribute amino groups to glycine during photorespiration (Liepman and Olsen, 2001; Novitskaya et al., 2002), and the extremophytes were able to maintain alanine levels under control and salt stress conditions whereas alanine content decreased in *Arabidopsis* in response to salt. Consistent with the idea that the extremophytes can sustain metabolic flux through the photorespiratory cycle, the glycine to serine ratio was always lower in the extremophytes than in *Arabidopsis*. A high glycine to serine ratio often correlates with the oxygenation of rubisco in the short term stages after induction of photorespiratory conditions (Novitskaya et al., 2002). However, increased flux through the photorespiratory pathway causes a reduction in the glycine to serine ratio (Timm et al., 2012). Further support for a higher photorespiratory flux in the extremophytes can be derived from the higher glycerate levels in *A. heirochuntic*a and *E. salsugineum* under control and salt stress conditions compared to *Arabidopsis*. Glycerate is the product of the penultimate reaction in the photorespiratory pathway and is converted to 3-phosphoglycerate, which is fed back into the Calvin cycle (Hagemann and Bauwe, 2016). As mentioned above, photorespiration plays an important role in excess energy dissipation in *A. hierochuntica* (Eppel et al., 2014).

Another reason for low levels of malate in *Arabidopsis* compared to the extremophytes might be due to the likely greater salt-mediated inhibition of photosynthesis in *Arabidopsis* (Stepien and Johnson, 2009). This could lead to reduced carbon flux through the glycolytic pathway and thus a need for the conversion of malate to pyruvate by malic enzyme to maintain the function of the TCA cycle (Casati et al., 1999). On the other hand, both *A. hierochuntica* and *E. salsugineum* exhibit a relatively higher photosynthetic rate and lower NPQ than *A. thaliana* and other plant species, under control and salt conditions, and under various CO_2_ concentrations and light intensities (**Figures 5G,H**; Stepien and Johnson, 2009; Eppel et al., 2014). Indeed, the higher overall levels of glucose, fructose, glucose 6-phosphate, and fructose 6-phosphate in the extremophytes may indicate a higher glycolysis flux either from photosynthesis or starch consumption, which may maintain pyruvate entry into the TCA cycle.

Raffinose and its precursor galactinol are thought to be osmoprotectants and antioxidants (Taji et al., 2002; Nishizawa et al., 2008; Farrant et al., 2009). We observed a large rise in galactinol and raffinose content in *Arabidopsis* in response to salt whereas levels of these metabolites remained low in the extremophytes (**Figure 8**). It is remarkable that extremophytes from two greatly differing habitats have both evolved to avoid accumulation of high levels of raffinose as a stress tolerance component similar to the desiccation-tolerant spike moss *Selaginella lepidophylla* (Yobi et al., 2012). Although, it is unclear what advantage is gained by not accumulating raffinose, this sugar can be metabolized into sucrose and thereafter into glucose and fructose for glycolysis.

The increased accumulation of metabolites observed in the extremophytes is costly (Munns, 2002) and could contribute to the slower growth rate of *E. salsugineum* compared to *Arabidopsis* (Lugan et al., 2010). However, *A. hierochuntica*, where the levels of most metabolites and therefore the costs are lower compared to *E. salsugineum*, exhibits fast germination and seedling establishment. It is possible that the saline habitat of *E. salsugineum*, demands constantly high levels of compatible solutes and antioxidants. In contrast, *A. hierochuntica* is exposed to lower salinity levels than *E. salsugineum*, but experiences intense light radiation, low water and nutrient availability and temperature extremes. By accumulating specific compounds, such as antioxidants, while moderately accumulating other solutes such as sugars and amino acids, *A. hierochuntica* could have developed a stress-tolerance strategy with lower metabolic costs, suited to its specific habitat, allowing fast seedling establishment and quick completion of its life cycle to avoid the dry season.

## Author Contributions

GE, RS, and SB conceived and designed the study. GE and RS performed all experiments except for the chromosome count, chlorophyll fluorescence and flow cytometry, which were performed by AK, AE, and AC, respectively, under the supervision of SB, SR, and NT-Z, respectively. GE and YK, under the supervision of AF, performed the metabolic profiling experiments and data analysis. TA participated in the oxidative stress experiments. YG supervised and participated in field collections of *A. hierochuntica* as well as advising on growth protocols. GE and SB wrote the article and all authors read and approved the manuscript.

## Conflict of Interest Statement

The authors declare that the research was conducted in the absence of any commercial or financial relationships that could be construed as a potential conflict of interest.

## Footnotes

1 http://www.ims.gov.il/IMSENG/All_Tahazit/homepage.htm

2 http://rsbweb.nih.gov/ij/
