# Supplementary material for: Anastatica hierochuntica, an Arabidopsis Desert Relative, Is Tolerant to Multiple Abiotic Stresses and Exhibits Species-Specific and Common Stress Tolerance Strategies with Its Halophytic Relative, Eutrema (Thellungiella) salsugineum
Source: Front Plant Sci. 2017 Jan 17;7:1992. doi: 10.3389/fpls.2016.01992 (PMC5239783; doi:10.3389/fpls.2016.01992)
Supplement: Supplementary file 1 [file Data_Sheet_1.PDF]

frontiers

Supplementary Material

Anastatica hierochuntica, an *Arabidopsis* Desert Relative, is Tolerant to Multiple Abiotic Stresses and Exhibits Species-Specific and Common Stress Tolerance Strategies with its Halophytic Relative, *Eutrema* (*Thellungiella*) *salsugineum*

Gil Eshel[1], Ruth Shaked[1], Yana Kazachkova[1], Asif Khan[1], Amir Eppel[1], Aroldo Cisneros[1], Tania Acuna[1], Yitzhak Gutterman[1], Noemi Tel-Zur[1], Shimon Rachmilevitch[1], Aaron Fait[1] and Simon Barak[1*]

[1]French Associates Institute for Biotechnology and Agriculture of Drylands, Jacob Blaustein Institutes for Desert Research, Ben-Gurion University of the Negev, Midreshet Ben-Gurion, Israel

*** Correspondence:**
Simon Barak, French Associates Institute for Biotechnology and Agriculture of Drylands, Jacob Blaustein Institutes for Desert Research, Ben-Gurion University of the Negev, Midreshet Ben-Gurion, 8499000, Israel

simon@bgu.ac.il

1 Supplementary Figures and Tables

The following supplementary figures and tables are included:
Supplementary Figure S1. Growth of *Arabidopsis* and *A. hierochuntica* under different light intensities.
Supplementary Figure S2. Effect of heat shock on growth parameters and pigment contents (absolute values) of *Arabidopsis* and *A. hierochuntica*.
Supplementary Figure S3. Effect of low NO_3^- concentrations on fresh weight (absolute values) of *Arabidopsis* and *A. hierochuntica*.
Supplementary Figure S4. The effect of salt stress on growth parameters (absolute values) of *Arabidopsis* and *A. hierochuntica*.
Supplementary Figure S5. The effect of oxidative stress on growth parameters and pigment contents (absolute values) of *Arabidopsis* and *A. hierochuntica*.
Supplementary Table S1. Absolute metabolite eigenvalues for the first two components of the PCA analysis of soil-grown plants under control and salt stress conditions.
Supplementary Table S2. Two-way ANOVA ($P < 0.05$) of metabolite response of soil-grown plants under control and salt stress conditions.
Supplementary Table S3. Ono-way ANOVA ($P < 0.05$) of metabolite response of soil-grown plants.

1.1 Supplementary Figures

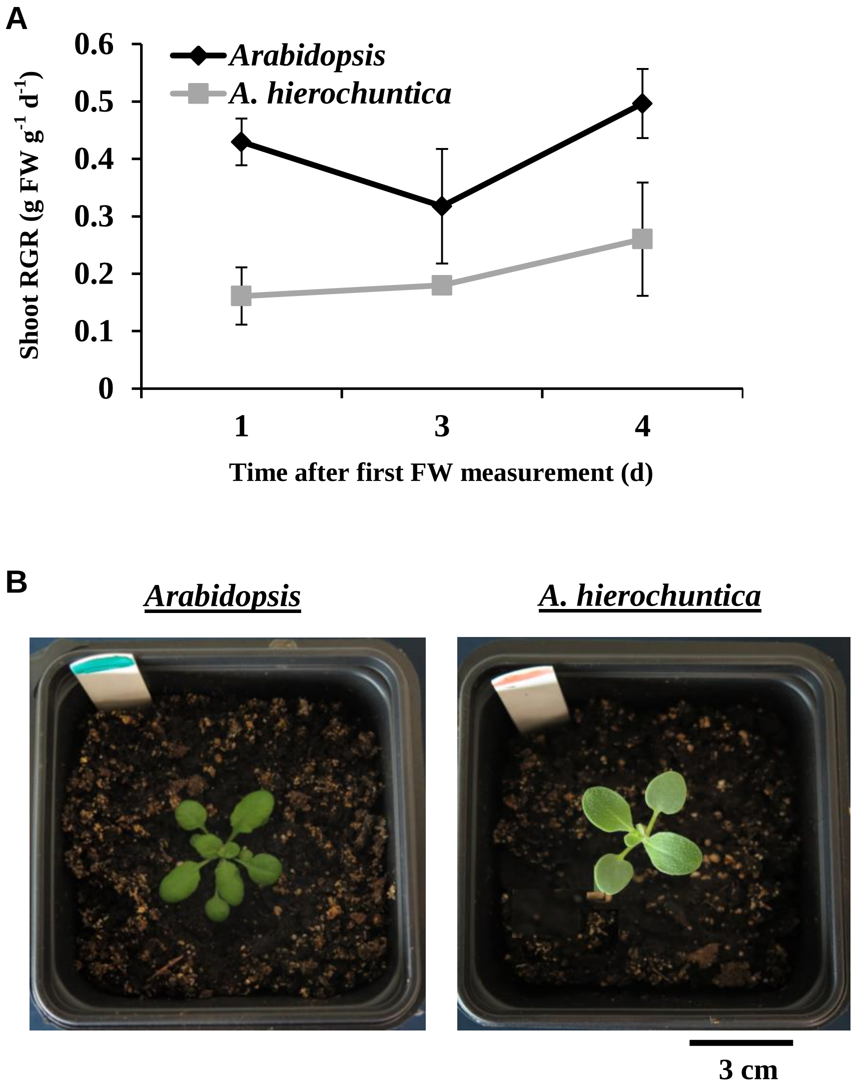

Supplementary Figure S1. Growth of *Arabidopsis* and *A. hierochuntica* under different light intensities. (A) Relative growth rate (RGR) of two-week old soil-grown plants grown under a light intensity of 250 µmol photons m^{-2} s^{-1}. RGR using fresh weight (FW) measurements was calculated according to Hunt (1990). Data are mean of RGR of 3 to 4 plants ± S.D. **(B)** Growth of plants under a light intensity of 350 µmol photons m^{-2} s^{-1}. The slower growth rate of *A. hierochuntica* can be discerned by the lower number of leaves produced compared to *Arabidopsis*.

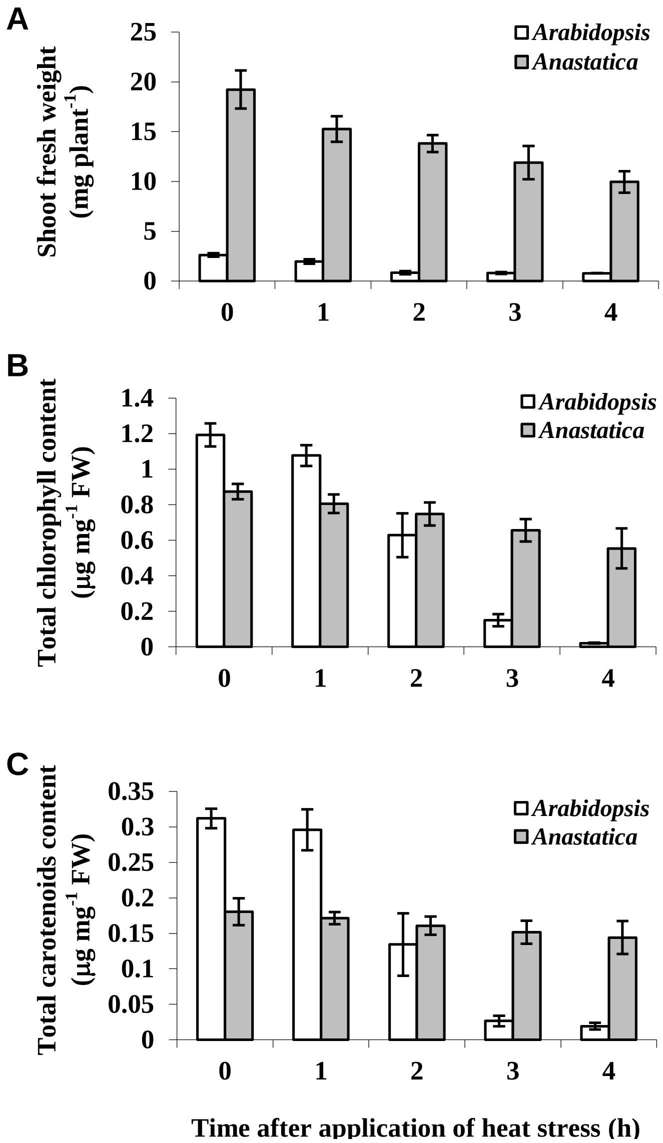

Supplementary Figure S2. Effect of heat shock on growth parameters and pigment contents (absolute values) of *Arabidopsis* and *A. hierochuntica*. Plant seedlings were grown on MS medium and harvested 48 h after exposure to 0, 1, 2, 3 or 4 h heat shock (45 ^{o}C). **(A)** Shoot fresh weight. **(B)** Total chlorophyll. **(C)** Total carotenoids. Data are mean of four independent experiments ± S.D. Each independent experiment comprised four replicates containing ca. 10 to 15 seedlings. FW, Fresh weight.

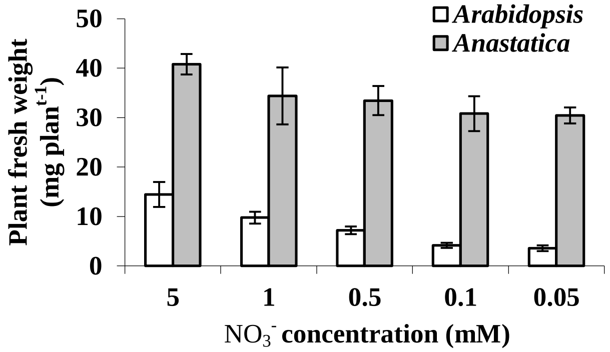

Supplementary Figure S3. Effect of low NO_3^- concentrations on fresh weight (absolute values) of *Arabidopsis* and *A. hierochuntica*. Plant seedlings were grown on MS medium with the indicated concentrations of KNO_3^-, and harvested after 6 d. **(A)** Shoot fresh weight. **(B)** Total chlorophyll. **(C)** Total carotenoids. Data are mean of three independent experiments ± S.D. Each independent experiment comprised four replicates containing ca. 10 to 15 seedlings. FW, Fresh weight.

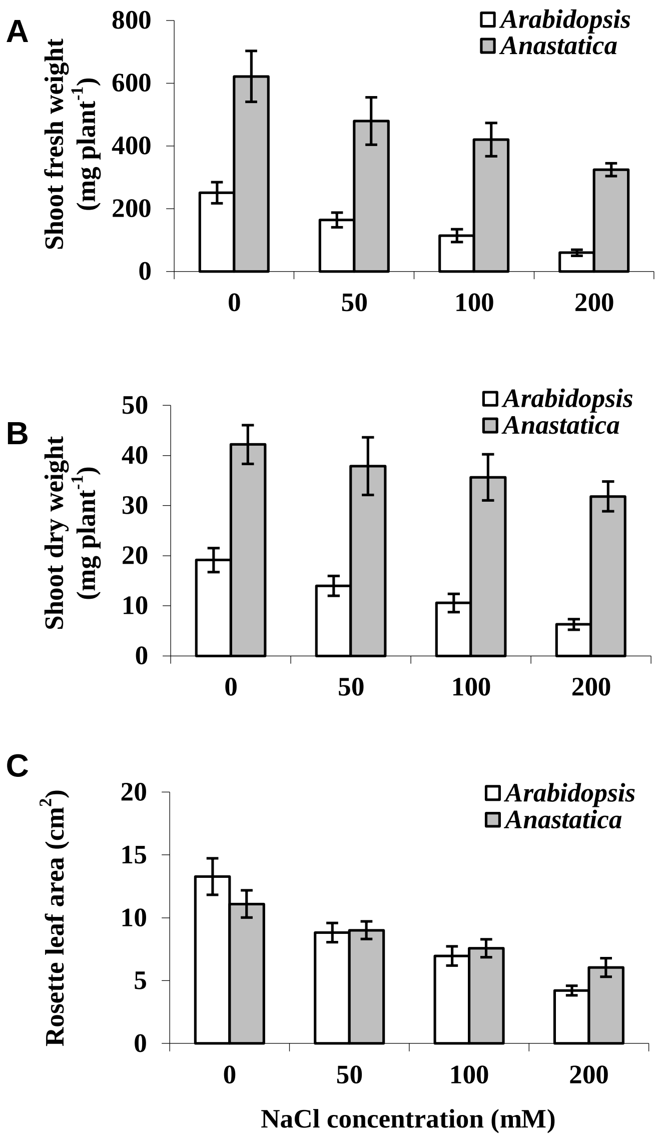

Supplementary Figure S4. The effect of salt stress on growth parameters (absolute values) of *Arabidopsis* and *A. hierochuntica*. Plants grown on soil were exposed to incremental increases of NaCl concentration, and were harvested one week after the final NaCl concentration was reached. (**A**) Shoot fresh weight. (**B**) Shoot dry weight. (**C**) Rosette leaf area. Data are mean of three independent experiments ± S.D. Each independent experiment comprised five replicate pots with 4 pooled plants per pot.

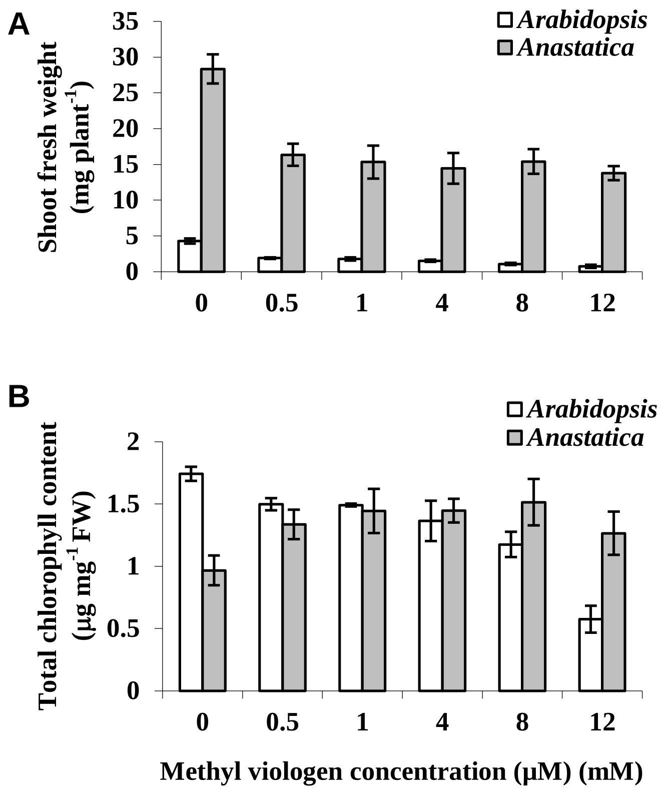

Supplementary Figure S5. The effect of oxidative stress on growth parameters and pigment contents (absolute values) of *Arabidopsis* and *A. hierochuntica*. Plant seedlings grown on vertical MS plates were transferred to 0, 0.5, 1, 4, 8 or 12 µM Methyl viologen for 5 d. **(A)** Shoot fresh weight. **(B)** Total chlorophyll content. Data are mean (n =4) ± SD. Each replicate plate contained ca. 10 (*Arabidopsis*) or ca. 6 (*Anastatica*) seedlings. Data are representative of two independent experiments. FW, Fresh weight.

Supplementary Table S1. Absolute metabolite eigenvalues for the first two components of the PCA analysis of soil-grown plants under control and salt stress conditions (Exp. 1)

Arabidopsis, E. salsugineum and A. hierochuntica

**Eigenvector 1**		**Eigenvector 2**	
**Metabolite name**	**Eigenvalue**	**Metabolite name**	**Eigenvalue**
Raffinose	2.77224	Proline	4.31851
Malate	2.50815	Citrate	2.52105
Galactinol	1.77843	Malate	1.9734
Urea	1.67861	Urea	1.70312
Citrate	1.26046	Glycine	1.29886
Fumarate	1.21338	Raffinose	1.29333
Phosphoric acid	1.20785	Serine	1.19845
Glycerate	1.145	Fumarate	1.18979
Glucose-6-phosphate	0.97274	Inositol, myo-	1.17031
Threonine, allo-	0.96323	Glycerophosphoglycerol	1.05007
Ascorbate	0.76112	Galactinol	0.86677
Alanine	0.75939	Pyroglutamic acid	0.85925
Fructose	0.73525	Threonine, allo-	0.85153
Thiazole, 4-methyl-5-hydroxyethyl-	0.69515	Sucrose	0.74691
Fructose-6-phosphate	0.65479	Aspartate	0.74289
Glycine	0.59395	Ascorbate	0.71526
Proline	0.52044	Glycerate	0.70461
Ribonic acid	0.41156	Glucose-6-phosphate	0.69377
Inositol, myo-	0.41098	Glucopyranose	0.67879
Dehydroascorbate	0.38639	Threonic acid	0.63017
Caffeic acid, trans-	0.36739	Valine	0.62136
Pyroglutamic acid	0.36436	Fructose-6-phosphate	0.42858
Aspartate	0.35871	Succinate	0.41608
Valine	0.32298	Dehydroascorbate	0.3878
Glucose	0.32057	Caffeic acid, trans-	0.36887
Ribose	0.28726	Phosphoric acid	0.35593
Threonic acid	0.23714	Ribose	0.26179
Serine	0.23137	Lactate	0.24707
Glucopyranose	0.22421	Glucose	0.21166
Succinate	0.20588	Ribonic acid	0.19662
Glycerophosphoglycerol	0.10872	Thiazole, 4-methyl-5-hydroxyethyl-	0.16486
Sucrose	0.09018	Fructose	0.15789
Lactate	0.06418	Alanine	0.13007

E. salsugineum and Anastatica

**Eigenvector 1**		**Eigenvector 2**	
**Metabolite name**	**Eigenvalue**	**Metabolite name**	**Eigenvalue**
Proline	4.38751	Fructose	2.90758
Urea	3.52414	Glycerophosphoglycerol	2.6836
Citrate	1.84853	Fumarate	2.05913
Glycerophosphoglycerol	1.6657	Citrate	1.45572
Inositol, myo-	1.46727	Galactinol	1.39092
Aspartate	1.45721	Inositol, myo-	1.24843
Raffinose	1.13922	Glycine	1.16098
Threonic acid	1.09677	Glucose	1.03164
Pyroglutamic acid	1.08713	Glucose-6-phosphate	0.99223
Threonine, allo-	1.08064	Raffinose	0.92317
Malate	0.96116	Glycerate	0.75553
Valine	0.82064	Urea	0.73008
Sucrose	0.78379	Fructose-6-phosphate	0.72801
Galactinol	0.68144	Pyroglutamic acid	0.725
Succinate	0.6525	Sucrose	0.68251
Glucose-6-phosphate	0.59484	Phosphoric acid	0.61967
Fumarate	0.57318	Glucopyranose	0.54672
Serine	0.5619	Valine	0.52424
Thiazole, 4-methyl-5-hydroxyethyl-	0.55204	Threonine, allo-	0.48647
Fructose-6-phosphate	0.54729	Thiazole, 4-methyl-5-hydroxyethyl-	0.45535
Ribonic acid	0.53273	Threonic acid	0.38343
Fructose	0.51495	Dehydroascorbate	0.35929
Ribose	0.44995	Ribonic acid	0.32315
Caffeic acid, trans-	0.42128	Ribose	0.30509
Glucopyranose	0.37208	Caffeic acid, trans-	0.24968
Alanine	0.32422	Aspartate	0.23083
Glycine	0.18869	Proline	0.22129
Lactate	0.16317	Malate	0.18064
Glycerate	0.15463	Succinate	0.15297
Dehydroascorbate	0.14705	Lactate	0.15196
Ascorbate	0.12663	Alanine	0.13683
Glucose	0.05861	Serine	0.06169
Phosphoric acid	0.03403	Ascorbate	0.0117

Supplementary Table S1. Absolute metabolite eigenvalues for the first two components of the PCA analysis of soil-grown plants under control and salt stress conditions (Exp. 2)

Arabidopsis, E. salsugineum and A. hierochuntica

Eigenvector 1		Eigenvector 2	
**Metabolite name**	**Eigenvalue**	**Metabolite name**	**Eigenvalue**
Raffinose (11TMS)	3.04917	Proline (1TMS)	4.22989
Proline [+CO2] (2TMS)	2.57678	Proline [+CO2] (2TMS)	3.47315
Aspartic acid (2TMS)	2.38117	Glycine (2TMS)	3.01859
Psicose (1MEOX) (5TMS) MP	2.22938	Citric acid (4TMS)	2.35359
Glucopyranose, D- (5TMS)	2.22793	Putrescine (4TMS)	1.57367
Mannose-6-phosphate (1MEOX) (6TMS) MP	2.05297	Threonine (3TMS)	1.54387
Psicose (1MEOX) (5TMS) BP	2.04598	Butanoic acid, 4-amino- (3TMS)	1.54295
Malic acid (3TMS)	2.00929	Raffinose (11TMS)	1.49921
Fumaric acid (2TMS)	1.87047	Inositol, myo- (6TMS)	1.23189
Phosphoric acid (3TMS)	1.81663	Glycine (3TMS)	1.04664
Fructose-6-phosphate (1MEOX) (6TMS) MP	1.67388	Proline (2TMS)	0.89252
Glucose-6-phosphate (1MEOX) (6TMS) BP	1.59892	Malonic acid (2TMS)	0.77434
Citric acid (4TMS)	1.56646	Glutamic acid (3TMS)	0.72756
Galactose (1MEOX) (5TMS) BP	1.54022	Erythronic acid (4TMS)	0.66417
Pyroglutamic acid (2TMS)	1.49914	Galactose (1MEOX) (5TMS) BP	0.62574
Butanoic acid, 4-amino- (3TMS)	1.48713	Glycerol-3-phosphate (4TMS)	0.60778
Glycerophosphoglycerol (5TMS)	1.34496	Malic acid (3TMS)	0.6003
Serine (4TMS)	1.33791	Fumaric acid (2TMS)	0.57112
Glucose (1MEOX) (5TMS) MP	1.32471	Asparagine (2TMS)	0.54031
Glutamic acid (3TMS)	1.23599	Galactinol (9TMS)	0.52991
Succinic acid (2TMS)	1.20487	Serine (3TMS)	0.50889
Glutamine [-H2O] (2TMS) BP	1.11438	Benzoic acid, (1TMS)	0.50439
Ascorbic acid (4TMS)	1.09366	Uracil (2TMS)	0.47859
Glyceric acid (3TMS)	1.08869	Arabinose (1MEOX) (4TMS) BP	0.45552
Galactinol (9TMS)	1.08684	Glucose (1MEOX) (5TMS) MP	0.43601
Glycine (2TMS)	1.05645	Sucrose (8TMS)	0.41393
Uracil (2TMS)	0.95263	Psicose (1MEOX) (5TMS) MP	0.40304
Proline (1TMS)	0.90776	Glycerophosphoglycerol (5TMS)	0.38371

Asparagine (2TMS)	0.83649	Glucopyranose, D- (5TMS)	0.38158
Serine (3TMS)	0.82234	Ascorbic acid (4TMS)	0.37133
Putrescine (4TMS)	0.80659	Psicose (1MEOX) (5TMS) BP	0.34429
Methionine (1TMS)	0.66878	Valine (2TMS)	0.33606
Benzoic acid, (1TMS)	0.64229	Threonic acid (4TMS)	0.31468
Erythronic acid (4TMS)	0.62956	Alanine (3TMS)	0.30601
Lyxose (1MEOX) (4TMS) MP	0.60317	Serine (4TMS)	0.28404
Glucopyranose [-H20] (4TMS)	0.56618	Glucose-6-phosphate (1MEOX) (6TMS) BP	0.27935
Alanine (2TMS)	0.52468	Phenylalanine (2TMS)	0.27773
Threonic acid (4TMS)	0.48966	Valine (1TMS)	0.26704
Dehydroascorbic acid dimer (2MEOX) MP	0.47044	Fructose-6-phosphate (1MEOX) (6TMS) MP	0.26398
Malonic acid (2TMS)	0.46751	Glucose, 1,6-anhydro, beta- (3TMS)	0.23384
Glycine (3TMS)	0.46095	Glycerol (3TMS)	0.21832
Glycerol (3TMS)	0.44736	Serine (2TMS)	0.20486
Threonine (3TMS)	0.43886	Ribonic acid (5TMS)	0.17427
Glucose, 1,6-anhydro, beta- (3TMS)	0.43363	Glyceric acid (3TMS)	0.17042
Threonic acid-1,4-lactone (2TMS)	0.41197	Methionine (1TMS)	0.1658
Valine (1TMS)	0.40729	Mannose-6-phosphate (1MEOX) (6TMS) MP	0.15828
Glycerol-3-phosphate (4TMS)	0.39213	Glutamic acid (2TMS)	0.14944
Alanine (3TMS)	0.36878	Threitol (4TMS)	0.14551
Sucrose (8TMS)	0.36302	Pyroglutamic acid (2TMS)	0.12582
Proline (2TMS)	0.34349	Aspartic acid (2TMS)	0.10978
Butyro-1,4-lactam (1TMS)	0.25688	Glutamine [-H2O] (2TMS) BP	0.08459
Threitol (4TMS)	0.22969	Butyro-1,4-lactam (1TMS)	0.08389
Valine (2TMS)	0.22871	Xylose, D- (1MEOX) (4TMS)	0.07794
Arabinose (1MEOX) (4TMS) BP	0.21977	Xylose (1MEOX) (4TMS) MP	0.07507
Ribonic acid (5TMS)	0.16647	Shikimic acid (4TMS)	0.05972
Serine (2TMS)	0.16483	Hexadecanoic acid (1TMS)	0.05459
Glutamic acid (2TMS)	0.15576	Dehydroascorbic acid dimer (2MEOX) MP	0.05211
Xylose, D- (1MEOX) (4TMS)	0.15534	Succinic acid (2TMS)	0.04775
Shikimic acid (4TMS)	0.11815	Glucopyranose [-H20] (4TMS)	0.04308
Phenylalanine (2TMS)	0.09349	Lyxose (1MEOX) (4TMS) MP	0.03694
Octadecanoic acid (1TMS)	0.08729	Phosphoric acid (3TMS)	0.03223
Inositol, myo- (6TMS)	0.079	Threonic acid-1,4-lactone (2TMS)	0.02355
Xylose (1MEOX) (4TMS) MP	0.04083	Alanine (2TMS)	0.01472

Hexadecanoic acid (1TMS)	0.00605	Octadecanoic acid (1TMS)	0.00181

E. salsugineum and Anastatica

**Eigenvector 1**		**Eigenvector 2**	
**Metabolite name**	**Eigenvalue**	**Metabolite name**	**Eigenvalue**
Psicose (1MEOX) (5TMS) MP	3.25081	Proline (1TMS)	4.98095
Dehydroascorbic acid dimer (2MEOX) MP	2.94219	Proline [+CO2] (2TMS)	3.35799
Glycine (2TMS)	2.92356	Citric acid (4TMS)	2.5723
Glycine (3TMS)	2.76551	Inositol, myo- (6TMS)	2.04975
Lyxose (1MEOX) (4TMS) MP	2.0934	Alanine (3TMS)	2.02897
Putrescine (4TMS)	2.06928	Glycerophosphoglycerol (5TMS)	2.01297
Glutamine [-H2O] (2TMS) BP	1.7599	Malic acid (3TMS)	1.72478
Fumaric acid (2TMS)	1.64618	Raffinose (11TMS)	1.70928
Fructose-6-phosphate (1MEOX) (6TMS) MP	1.59787	Threonic acid (4TMS)	1.70444
Valine (1TMS)	1.57386	Serine (4TMS)	1.66766
Glucopyranose [-H20] (4TMS)	1.55221	Threonine (3TMS)	1.60544
Mannose-6-phosphate (1MEOX) (6TMS) MP	1.50082	Glycerol-3-phosphate (4TMS)	1.519
Asparagine (2TMS)	1.43896	Glycine (2TMS)	1.48954
Glucose-6-phosphate (1MEOX) (6TMS) BP	1.42184	Valine (2TMS)	1.37474
Glutamic acid (3TMS)	1.19215	Glucopyranose, D- (5TMS)	1.32293
Xylose (1MEOX) (4TMS) MP	1.18103	Succinic acid (2TMS)	1.3226
Malic acid (3TMS)	1.10727	Lyxose (1MEOX) (4TMS) MP	1.31951
Proline [+CO2] (2TMS)	1.09202	Valine (1TMS)	1.24375
Threonic acid (4TMS)	1.0455	Fumaric acid (2TMS)	1.20678
Proline (1TMS)	1.03034	Glycine (3TMS)	1.15348
Galactinol (9TMS)	1.02339	Dehydroascorbic acid dimer (2MEOX) MP	1.0961
Glycerophosphoglycerol (5TMS)	0.892	Serine (3TMS)	1.05685
Pyroglutamic acid (2TMS)	0.88272	Psicose (1MEOX) (5TMS) MP	1.02628
Galactose (1MEOX) (5TMS) BP	0.84367	Ascorbic acid (4TMS)	1.02415
Glucose, 1,6-anhydro, beta- (3TMS)	0.80845	Mannose-6-phosphate (1MEOX) (6TMS) MP	1.01166
Glucopyranose, D- (5TMS)	0.78079	Glutamine [-H2O] (2TMS) BP	1.00113
Psicose (1MEOX) (5TMS) BP	0.77938	Fructose-6-phosphate (1MEOX)	0.94591

		(6TMS) MP	
Alanine (3TMS)	0.76472	Glyceric acid (3TMS)	0.93159
Ascorbic acid (4TMS)	0.7385	Glutamic acid (3TMS)	0.9163
Proline (2TMS)	0.72416	Erythronic acid (4TMS)	0.90493
Methionine (1TMS)	0.67099	Phosphoric acid (3TMS)	0.85209
Arabinose (1MEOX) (4TMS) BP	0.66566	Pyroglutamic acid (2TMS)	0.84534
Erythronic acid (4TMS)	0.60619	Sucrose (8TMS)	0.80513
Threonic acid-1,4-lactone (2TMS)	0.60047	Glucose-6-phosphate (1MEOX) (6TMS) BP	0.80124
Aspartic acid (2TMS)	0.56476	Galactinol (9TMS)	0.67925
Ribonic acid (5TMS)	0.55236	Threonic acid-1,4-lactone (2TMS)	0.63577
Glucose (1MEOX) (5TMS) MP	0.53216	Psicose (1MEOX) (5TMS) BP	0.61025
Citric acid (4TMS)	0.53027	Glucose, 1,6-anhydro, beta- (3TMS)	0.6066
Glyceric acid (3TMS)	0.50051	Xylose (1MEOX) (4TMS) MP	0.56764
Threonine (3TMS)	0.48285	Aspartic acid (2TMS)	0.5293
Raffinose (11TMS)	0.46045	Glycerol (3TMS)	0.51716
Butyro-1,4-lactam (1TMS)	0.45578	Uracil (2TMS)	0.49054
Threitol (4TMS)	0.44918	Malonic acid (2TMS)	0.42607
Succinic acid (2TMS)	0.33992	Ribonic acid (5TMS)	0.41756
Inositol, myo- (6TMS)	0.32827	Glucose (1MEOX) (5TMS) MP	0.39032
Sucrose (8TMS)	0.26823	Putrescine (4TMS)	0.38494
Glutamic acid (2TMS)	0.25002	Serine (2TMS)	0.37401
Serine (4TMS)	0.22051	Glutamic acid (2TMS)	0.35781
Benzoic acid, (1TMS)	0.20003	Proline (2TMS)	0.35143
Butanoic acid, 4-amino- (3TMS)	0.16862	Shikimic acid (4TMS)	0.3268
Shikimic acid (4TMS)	0.15581	Galactose (1MEOX) (5TMS) BP	0.29135
Valine (2TMS)	0.15475	Butanoic acid, 4-amino- (3TMS)	0.23501
Phosphoric acid (3TMS)	0.14978	Phenylalanine (2TMS)	0.22598
Glycerol (3TMS)	0.11589	Glucopyranose [-H20] (4TMS)	0.17855
Xylose, D- (1MEOX) (4TMS)	0.11209	Xylose, D- (1MEOX) (4TMS)	0.17707
Phenylalanine (2TMS)	0.10236	Alanine (2TMS)	0.16935
Malonic acid (2TMS)	0.09691	Methionine (1TMS)	0.16682
Serine (2TMS)	0.08434	Arabinose (1MEOX) (4TMS) BP	0.15184
Glycerol-3-phosphate (4TMS)	0.08224	Asparagine (2TMS)	0.03692
Uracil (2TMS)	0.06213	Butyro-1,4-lactam (1TMS)	0.0318
Hexadecanoic acid (1TMS)	0.06036	Octadecanoic acid (1TMS)	0.03032
Alanine (2TMS)	0.05348	Benzoic acid, (1TMS)	0.02425
Octadecanoic acid (1TMS)	0.04891	Threitol (4TMS)	0.00824
Serine (3TMS)	0.034	Hexadecanoic acid (1TMS)	0.00226

Supplementary Table S2. Two-way ANOVA (*P* < 0.05) of metabolite response of soil-grown plants under control and salt stress conditions (Exp. 1)

Arabidopsis, E. salsugineum and A. heirochuntica

Species significant metabolites	F-ratio	Treatment (salt) significant metabolites	F-ratio	Species x Treatment significant metabolites	F-ratio
Valine (2TMS)	26.94	Valine (2TMS)	16.19	Valine (2TMS)	2.95
Serine (2TMS)	7.83	Raffinose (11TMS)	49.76	Serine (2TMS)	50.44
Phosphoric acid (3TMS)	54.84	Pyroglutamic acid (2TMS)	6.38	Phosphoric acid (3TMS)	8.08
Succinic acid (2TMS)	26.79	Malic acid (3TMS)	22.96	Glyceric acid (3TMS)	10.04
Glyceric acid (3TMS)	45.82	Galactinol (9TMS)	31.99	Fumaric acid (2TMS)	17.19
Fumaric acid (2TMS)	71.43	Fumaric acid (2TMS)	21.57	Alanine (3TMS)	31.19
Alanine (3TMS)	52.04	Threonine, allo- (3TMS)	33.19	Threonine, allo- (3TMS)	13.66
Threonine, allo- (3TMS)	57.44	Alanine (3TMS)	7.72	Malic acid (3TMS)	15.90
Malic acid (3TMS)	97.98	Fructose-6-phosphate (1MEOX) (6TMS) MP	5.30	Threonic acid (4TMS)	9.34
Pyroglutamic acid (2TMS)	122.61	Glyceric acid (3TMS)	8.96	Proline (pooled)	10.59
Threonic acid (4TMS)	40.80	Threonic acid (4TMS)	10.23	Ribonic acid (5TMS)	5.96
Proline	37.92	Proline (pooled)	208.47	Glucopyranose [-H20] (4TMS)	5.06
Ribose (1MEOX) (4TMS) BP	11.02	Glucopyranose [-H20] (4TMS)	9.52	Glucose (1MEOX) (5TMS) MP	3.48
Ribonic acid (5TMS)	19.35	Glucose (1MEOX) (5TMS) MP	6.30	Fructose-6-phosphate (1MEOX) (6TMS) MP	12.08
Glucopyranose [-H20] (4TMS)	32.19	Valine (2TMS)	17.74	Glucose-6-phosphate (1MEOX) (6TMS) BP	7.98
Fructose (1MEOX) (5TMS) BP	134.50	Succinic acid (2TMS)	11.72	Sucrose (8TMS)	3.09
Dehydroascorbic acid dimer (2MEOX) MP	28.30	Ribonic acid (5TMS)	5.06	Galactinol (9TMS)	23.62
Glucose (1MEOX) (5TMS) MP	27.49	Sucrose (8TMS)	13.53	Raffinose (11TMS)	51.86
Ascorbic acid (4TMS)	49.94	Ribose (1MEOX) (4TMS) BP	3.94	Glycerophosphoglycerol (5TMS)	9.00
Inositol, myo- (6TMS)	4.01	Serine (2TMS)	44.33		
Fructose-6-phosphate (1MEOX) (6TMS) MP	56.57	Inositol, myo- (6TMS)	59.85		
Glucose-6-phosphate (1MEOX) (6TMS) BP	84.31	Lactic acid, DL- (2TMS)	3.57		
Sucrose (8TMS)	17.77	Glycerophosphoglycerol (5TMS)	27.99		
Galactinol (9TMS)	84.95				
Raffinose (11TMS)	139.85				
Glycerophosphoglycerol (5TMS)	209.55				

Arabidopsis* and *E. salsugineum

Species significant metabolites	F-ratio	Treatment (salt) significant metabolites	F-ratio	Species x Treatment significant metabolites	F-ratio
Urea (2TMS)	81.90	Valine (2TMS)	12.46	Urea (2TMS)	10.39
Serine (2TMS)	7.16	Urea (2TMS)	61.45	Serine (2TMS)	102.01
Phosphoric acid (3TMS)	150.10	Serine (2TMS)	36.17	Succinic acid (2TMS)	4.31
Glycine (3TMS)	14.65	Phosphoric acid (3TMS)	10.76	Glyceric acid (3TMS)	16.49
Succinic acid (2TMS)	52.80	Glycine (3TMS)	23.21	Fumaric acid (2TMS)	16.33
Glyceric acid (3TMS)	92.88	Succinic acid (2TMS)	7.06	Alanine (3TMS)	37.53
Fumaric acid (2TMS)	55.53	Glyceric acid (3TMS)	15.57	Threonine, allo- (3TMS)	17.82
Alanine (3TMS)	63.74	Fumaric acid (2TMS)	21.11	Glutamine [-H2O] (2TMS) BP	16.00
Threonine, allo- (3TMS)	57.44	Alanine (3TMS)	20.08	Malic acid (3TMS)	21.07
Glutamine [-H2O] (2TMS) BP	43.00	Threonine, allo- (3TMS)	23.17	Threonic acid (4TMS)	8.73
Malic acid (3TMS)	149.71	Glutamine [-H2O] (2TMS) BP	7.03	Proline (pooled)	10.89
Pyroglutamic acid (2TMS)	212.29	Malic acid (3TMS)	19.68	Ribose (1MEOX) (4TMS) BP	3.81
Threonic acid (4TMS)	43.64	Threonic acid (4TMS)	8.67	Ribonic acid (5TMS)	8.60
Proline (pooled)	18.56	Proline (pooled)	114.72	Glucopyranose [-H20] (4TMS)	4.12
Ribose (1MEOX) (4TMS) BP	19.96	Glucopyranose [-H20] (4TMS)	11.23	Fructose-6-phosphate (1MEOX) (6TMS) MP	9.24
Ribonic acid (5TMS)	30.30	Fructose (1MEOX) (5TMS) BP	6.05	Glucose-6-phosphate (1MEOX) (6TMS) BP	11.57
Glucopyranose [-H20] (4TMS)	46.73	Inositol, myo- (6TMS)	34.65	Galactinol (9TMS)	23.44
Fructose (1MEOX) (5TMS) BP	190.00	Fructose-6-phosphate (1MEOX) (6TMS) MP	7.88	Raffinose (11TMS)	55.38
Dehydroascorbic acid dimer (2MEOX) MP	49.51	Glucose-6-phosphate (1MEOX) (6TMS) BP	8.20	Glycerophosphoglycerol (5TMS)	12.86
Glucose (1MEOX) (5TMS) MP	36.77	Sucrose (8TMS)	13.20		
Ascorbic acid (4TMS)	87.65	Galactinol (9TMS)	27.45		
Fructose-6-phosphate (1MEOX) (6TMS) MP	67.79	Raffinose (11TMS)	50.82		
Glucose-6-phosphate (1MEOX) (6TMS) BP	188.36	Glycerophosphoglycerol (5TMS)	18.71		
Sucrose (8TMS)	6.97				
Galactinol (9TMS)	84.70				
Raffinose (11TMS)	142.27				
Glycerophosphoglycerol (5TMS)	193.45				

Arabidopsis and Anastatica

Species significant metabolites	F-ratio	Treatment (salt) significant metabolites	F-ratio	Species x Treatment significant metabolites	F-ratio
Valine (2TMS)	54.15	Valine (2TMS)	12.45	Valine (2TMS)	3.05
Serine (2TMS)	11.85	Serine (2TMS)	83.16	Serine (2TMS)	25.89
Phosphoric acid (3TMS)	108.22	Phosphoric acid (3TMS)	5.13	Phosphoric acid (3TMS)	9.39
Succinic acid (2TMS)	9.82	Succinic acid (2TMS)	5.61	Succinic acid (2TMS)	9.78
Glyceric acid (3TMS)	155.70	Fumaric acid (2TMS)	17.03	Glyceric acid (3TMS)	3.55
Fumaric acid (2TMS)	167.08	Alanine (3TMS)	21.53	Fumaric acid (2TMS)	16.13
Alanine (3TMS)	78.91	Threonine, allo- (3TMS)	29.50	Alanine (3TMS)	39.17
Threonine, allo- (3TMS)	88.59	Pyroglutamic acid (2TMS)	5.99	Threonine, allo- (3TMS)	9.78
Malic acid (3TMS)	169.17	Threonic acid (4TMS)	4.36	Pyroglutamic acid (2TMS)	4.14
Pyroglutamic acid (2TMS)	13.22	Proline (pooled)	199.47	Threonic acid (4TMS)	11.08
Proline (pooled)	17.21	Glucopyranose [-H20] (4TMS)	7.52	Proline (pooled)	5.55
Ribose (1MEOX) (4TMS) BP	11.94	Fructose (1MEOX) (5TMS) BP	16.18	Glucopyranose [-H20] (4TMS)	7.91
Ribonic acid (5TMS)	28.25	Glucose (1MEOX) (5TMS) MP	11.49	Fructose (1MEOX) (5TMS) BP	7.75
Glucopyranose [-H20] (4TMS)	4.45	Inositol, myo- (6TMS)	29.76	Dehydroascorbic acid dimer (2MEOX) MP	3.51
Fructose (1MEOX) (5TMS) BP	23.00	Fructose-6-phosphate (1MEOX) (6TMS) MP	18.63	Glucose (1MEOX) (5TMS) MP	3.13
Dehydroascorbic acid dimer (2MEOX) MP	39.41	Sucrose (8TMS)	11.86	Fructose-6-phosphate (1MEOX) (6TMS) MP	16.48
Glucose (1MEOX) (5TMS) MP	4.71	Galactinol (9TMS)	36.49	Sucrose (8TMS)	9.31
Ascorbic acid (4TMS)	127.60	Raffinose (11TMS)	59.26	Galactinol (9TMS)	29.08
Inositol, myo- (6TMS)	16.33	Glycerophosphoglycerol (5TMS)	18.11	Raffinose (11TMS)	57.61
Fructose-6-phosphate (1MEOX) (6TMS) MP	261.11				
Glucose-6-phosphate (1MEOX) (6TMS) BP	74.67				
Sucrose (8TMS)	11.77				
Galactinol (9TMS)	97.88				
Raffinose (11TMS)	144.75				
Glycerophosphoglycerol (5TMS)	17.73				

Eutrema and Anastatica

Species significant metabolites	F-ratio	Treatment (salt) significant metabolites	F-ratio	Species x Treatment significant metabolites	F-ratio
Valine (2TMS)	96.43	Valine (2TMS)	9.96	Serine (2TMS)	22.19
Phosphoric acid (3TMS)	13.21	Succinic acid (2TMS)	11.27	Phosphoric acid (3TMS)	8.93
Succinic acid (2TMS)	9.16	Glyceric acid (3TMS)	9.77	Glyceric acid (3TMS)	9.95
Fumaric acid (2TMS)	85.69	Fumaric acid (2TMS)	4.16	Fumaric acid (2TMS)	3.65
Aspartic acid (2TMS)	115.26	Alanine (3TMS)	10.79	Alanine (3TMS)	6.63
Malic acid (3TMS)	45.69	Threonine, allo- (3TMS)	14.99	"Threonine, allo-	5.92

				(3TMS)"	
Pyroglutamic acid (2TMS)	115.36	Aspartic acid (2TMS)	53.71	Aspartic acid (2TMS)	37.35
Threonic acid (4TMS)	46.16	Malic acid (3TMS)	25.42	Malic acid (3TMS)	13.90
Proline (pooled)	97.41	Pyroglutamic acid (2TMS)	5.03	Threonic acid (4TMS)	7.62
Ribose (1MEOX) (4TMS) BP	4.95	Glutamic acid (2TMS)	13.03	Proline (pooled)	16.57
Glucopyranose [-H20] (4TMS)	33.03	Threonic acid (4TMS)	13.19	Ribonic acid (5TMS)	5.30
Citric acid (4TMS)	7.99	Proline (pooled)	138.09	Citric acid (4TMS)	9.67
Fructose (1MEOX) (5TMS) BP	113.67	Ribose (1MEOX) (4TMS) BP	5.50	Glucose (1MEOX) (5TMS) MP	5.54
Glucose (1MEOX) (5TMS) MP	26.47	Ribonic acid (5TMS)	9.64	Fructose-6-phosphate (1MEOX) (6TMS) MP	19.23
Fructose-6-phosphate (1MEOX) (6TMS) MP	15.77	Citric acid (4TMS)	49.63	Glucose-6-phosphate (1MEOX) (6TMS) BP	9.81
Glucose-6-phosphate (1MEOX) (6TMS) BP	32.56	Fructose (1MEOX) (5TMS) BP	13.95	Sucrose (8TMS)	5.03
Sucrose (8TMS)	30.48	Glucose (1MEOX) (5TMS) MP	3.87	Galactinol (9TMS)	3.91
Galactinol (9TMS)	10.49	Inositol, myo- (6TMS)	53.76	Raffinose (11TMS)	173.00
Raffinose (11TMS)	153.69	Fructose-6-phosphate (1MEOX) (6TMS) MP	8.18	Caffeic acid, trans- (3TMS)	21.78
Caffeic acid, trans- (3TMS)	23.16	Glucose-6-phosphate (1MEOX) (6TMS) BP	3.91	Glycerophosphoglycerol (5TMS)	6.57
Glycerophosphoglycerol (5TMS)	560.54	Sucrose (8TMS)	6.72		
		Galactinol (9TMS)	37.95		
		Raffinose (11TMS)	62.69		
		Caffeic acid, trans- (3TMS)	19.90		
		Glycerophosphoglycerol (5TMS)	8.83		

Supplementary Table S2. Two-way ANOVA (*P*< 0.05) of metabolite response of soil-grown plants under control and salt stress conditions (Exp. 2)

Arabidopsis, E. salsugineum and A. hierochuntica

Species significant metabolites	F-ratio	Treatment (salt) significant metabolites	F-ratio	Species x Treatment significant metabolites	F-ratio
Valine (1TMS)	188.52	Valine (1TMS)	18.95	Alanine (2TMS)	4.25
Alanine (2TMS)	11.88	Glycine (2TMS)	48.28	Butyro-1,4-lactam (1TMS)	3.58
Glycine (2TMS)	30.69	Butyro-1,4-lactam (1TMS)	3.91	Valine (2TMS)	46.05
Butyro-1,4-lactam (1TMS)	15.65	Proline (1TMS)	91.59	Benzoic acid, (1TMS)	10.36
Proline (1TMS)	6.09	Valine (2TMS)	34.73	Serine (2TMS)	14.73
Benzoic acid, (1TMS)	35.82	Benzoic acid, (1TMS)	4.67	Phosphoric acid (3TMS)	7.84
Glycerol (3TMS)	36.43	Glycerol (3TMS)	12.05	Proline (2TMS)	39.81
Phosphoric acid (3TMS)	85.34	Proline (2TMS)	59.13	Glycine (3TMS)	8.17
Proline (2TMS)	30.38	Glycine (3TMS)	13.66	Succinic acid (2TMS)	14.80
Glycine (3TMS)	76.89	Uracil (2TMS)	7.84	Glyceric acid (3TMS)	8.45
Succinic acid (2TMS)	40.46	Serine (3TMS)	32.31	Uracil (2TMS)	19.87
Glyceric acid (3TMS)	32.86	Threonine (3TMS)	107.09	Fumaric acid (2TMS)	10.63
Uracil (2TMS)	15.74	Malic acid (3TMS)	3.84	Serine (3TMS)	24.05
Fumaric acid (2TMS)	458.58	Pyroglutamic acid (2TMS)	3.83	Alanine (3TMS)	3.76
Serine (3TMS)	60.85	Erythronic acid (4TMS)	12.53	Threonic acid-1,4-lactone (2TMS)	3.26
Alanine (3TMS)	6.41	Threonic acid (4TMS)	6.20	Threonine (3TMS)	22.75
Threonic acid-1,4-lactone (2TMS)	27.82	Serine (4TMS)	4.42	Methionine (1TMS)	7.71
Threonine (3TMS)	12.77	Proline [+CO2] (2TMS)	52.03	Aspartic acid (2TMS)	5.64
Methionine (1TMS)	43.19	Arabinose (1MEOX) (4TMS) BP	28.04	Malic acid (3TMS)	6.01
Aspartic acid (2TMS)	28.08	Glycerol-3-phosphate (4TMS)	17.46	Threitol (4TMS)	3.65
Malic acid (3TMS)	71.68	Citric acid (4TMS)	13.74	Pyroglutamic acid (2TMS)	14.33
Threitol (4TMS)	24.86	Psicose (1MEOX) (5TMS) BP	15.71	Serine (4TMS)	61.69
Pyroglutamic acid (2TMS)	140.20	Psicose (1MEOX) (5TMS) MP	25.08	Arabinose (1MEOX) (4TMS) BP	6.05
Erythronic acid (4TMS)	30.65	Glucose (1MEOX) (5TMS) MP	8.33	Glucose, 1,6-anhydro, beta- (3TMS)	4.50
Threonic acid (4TMS)	26.13	Galactose (1MEOX) (5TMS) BP	21.24	Glycerol-3-phosphate (4TMS)	14.26
Serine (4TMS)	165.11	Inositol, myo- (6TMS)	49.59	Shikimic acid (4TMS)	3.66
Proline [+CO2] (2TMS)	45.41	Glycerophosphoglycerol (5TMS)	4.57	Psicose (1MEOX) (5TMS) BP	5.53
Xylose (1MEOX) (4TMS) MP	61.70	Fructose-6-phosphate (1MEOX) (6TMS) MP	7.95	Psicose (1MEOX) (5TMS) MP	5.25
Lyxose (1MEOX) (4TMS) MP	139.15	Glucose-6-phosphate (1MEOX) (6TMS) BP	4.91	Glucose (1MEOX) (5TMS) MP	7.85
Arabinose (1MEOX) (4TMS) BP	38.89	Sucrose (8TMS)	17.44	Galactose (1MEOX) (5TMS) BP	10.28

Glucose, 1,6-anhydro, beta- (3TMS)	23.13	Galactinol (9TMS)	8.61	Inositol, myo- (6TMS)	5.55
Glycerol-3-phosphate (4TMS)	8.51	Raffinose (11TMS)	138.36	Glycerophosphoglycerol (5TMS)	8.57
Glucopyranose [-H20] (4TMS)	28.00			Fructose-6-phosphate (1MEOX) (6TMS) MP	18.48
Citric acid (4TMS)	6.90			Mannose-6-phosphate (1MEOX) (6TMS) MP	5.44
Psicose (1MEOX) (5TMS) BP	560.40			Glucose-6-phosphate (1MEOX) (6TMS) BP	4.15
Dehydroascorbic acid dimer (2MEOX) MP	39.03			Sucrose (8TMS)	6.03
Psicose (1MEOX) (5TMS) MP	838.10			Galactinol (9TMS)	7.35
Glucose (1MEOX) (5TMS) MP	79.70			Raffinose (11TMS)	70.18
Galactose (1MEOX) (5TMS) BP	131.75				
Ascorbic acid (4TMS)	45.46				
Glucopyranose, D- (5TMS)	25.02				
Inositol, myo- (6TMS)	3.79				
Glycerophosphoglycerol (5TMS)	82.79				
Octadecanoic acid (1TMS)	4.88				
Fructose-6-phosphate (1MEOX) (6TMS) MP	360.35				
Mannose-6-phosphate (1MEOX) (6TMS) MP	136.45				
Glucose-6-phosphate (1MEOX) (6TMS) BP	114.70				
Sucrose (8TMS)	39.80				
Galactinol (9TMS)	115.26				
Raffinose (11TMS)	603.76				

Arabidopsis and E. salsugineum

**Species significant metabolites**	**F-ratio**	**Treatment (salt) significant metabolites**	**F-ratio**	**Species x Treatment significant metabolites**	**F-ratio**
Alanine (2TMS)	19.74	Valine (1TMS)	18.87	Valine (2TMS)	19.38
Glycine (2TMS)	304.06	Glycine (2TMS)	344.58	Benzoic acid, (1TMS)	20.12
Butyro-1,4-lactam (1TMS)	17.57	Proline (1TMS)	271.79	Serine (2TMS)	18.73
Proline (1TMS)	105.72	Valine (2TMS)	10.04	Phosphoric acid (3TMS)	42.71
Valine (2TMS)	5.37	Benzoic acid, (1TMS)	22.79	Proline (2TMS)	38.87
Benzoic acid, (1TMS)	107.36	Serine (2TMS)	12.26	Glycine (3TMS)	11.21
Glycerol (3TMS)	21.19	Phosphoric acid (3TMS)	17.72	Succinic acid (2TMS)	107.08
Phosphoric acid (3TMS)	509.22	Proline (2TMS)	12.52	Glyceric acid (3TMS)	121.97
Proline (2TMS)	58.48	Glycine (3TMS)	162.93	Uracil (2TMS)	78.42
Glycine (3TMS)	428.29	Glyceric acid (3TMS)	25.01	Fumaric acid (2TMS)	6.57

Succinic acid (2TMS)	323.43	Uracil (2TMS)	40.59	Serine (3TMS)	7.43
Glyceric acid (3TMS)	596.19	Fumaric acid (2TMS)	5.38	Alanine (3TMS)	7.29
Uracil (2TMS)	81.64	Serine (3TMS)	4.65	Threonic acid-1,4-lactone (2TMS)	8.63
Fumaric acid (2TMS)	203.53	Threonine (3TMS)	25.48	Threonine (3TMS)	11.79
Serine (3TMS)	146.11	Aspartic acid (2TMS)	211.70	Aspartic acid (2TMS)	169.71
Alanine (3TMS)	14.13	Malic acid (3TMS)	17.41	Malic acid (3TMS)	65.58
Threonic acid-1,4-lactone (2TMS)	75.33	Pyroglutamic acid (2TMS)	18.95	Threitol (4TMS)	4.13
Threonine (3TMS)	28.65	Butanoic acid, 4-amino- (3TMS)	13.66	Pyroglutamic acid (2TMS)	35.36
Aspartic acid (2TMS)	1131.42	Erythronic acid (4TMS)	47.46	Butanoic acid, 4-amino- (3TMS)	27.97
Malic acid (3TMS)	843.07	Threonic acid (4TMS)	11.85	Erythronic acid (4TMS)	18.92
Threitol (4TMS)	67.92	Serine (4TMS)	13.31	Threonic acid (4TMS)	10.47
Pyroglutamic acid (2TMS)	462.27	Proline [+CO2] (2TMS)	252.79	Serine (4TMS)	21.32
Butanoic acid, 4-amino- (3TMS)	17.76	Xylose (1MEOX) (4TMS) MP	8.19	Proline [+CO2] (2TMS)	8.58
Erythronic acid (4TMS)	279.71	Arabinose (1MEOX) (4TMS) BP	5.07	Glucose, 1,6-anhydro, beta- (3TMS)	23.47
Threonic acid (4TMS)	105.45	Citric acid (4TMS)	234.78	Shikimic acid (4TMS)	15.50
Serine (4TMS)	274.44	Psicose (1MEOX) (5TMS) BP	15.00	Citric acid (4TMS)	15.82
Proline [+CO2] (2TMS)	738.75	Psicose (1MEOX) (5TMS) MP	20.42	Psicose (1MEOX) (5TMS) BP	7.98
Xylose (1MEOX) (4TMS) MP	47.49	Glucose (1MEOX) (5TMS) MP	28.75	Psicose (1MEOX) (5TMS) MP	6.46
Xylose, D- (1MEOX) (4TMS)	8.88	Galactose (1MEOX) (5TMS) BP	31.06	Glucose (1MEOX) (5TMS) MP	39.34
Arabinose (1MEOX) (4TMS) BP	15.42	Ascorbic acid (4TMS)	8.79	Galactose (1MEOX) (5TMS) BP	66.43
Glycerol-3-phosphate (4TMS)	20.63	Glucopyranose, D- (5TMS)	15.39	Ascorbic acid (4TMS)	9.21
Glucopyranose [-H20] (4TMS)	85.06	Inositol, myo- (6TMS)	126.29	Glucopyranose, D- (5TMS)	46.16
Citric acid (4TMS)	313.60	Fructose-6-phosphate (1MEOX) (6TMS) MP	34.83	Inositol, myo- (6TMS)	16.83
Psicose (1MEOX) (5TMS) BP	1801.06	Mannose-6-phosphate (1MEOX) (6TMS) MP	31.46	Glycerophosphoglycerol (5TMS)	23.35
Dehydroascorbic acid dimer (2MEOX) MP	78.92	Glucose-6-phosphate (1MEOX) (6TMS) BP	30.51	Fructose-6-phosphate (1MEOX) (6TMS) MP	12.77
Psicose (1MEOX) (5TMS) MP	2551.31	Sucrose (8TMS)	16.97	Mannose-6-phosphate (1MEOX) (6TMS) MP	16.71
Glucose (1MEOX) (5TMS) MP	419.00	Galactinol (9TMS)	12.68	Glucose-6-phosphate (1MEOX) (6TMS) BP	13.31
Galactose (1MEOX) (5TMS) BP	644.64	Raffinose (11TMS)	78.55	Galactinol (9TMS)	10.32
Ascorbic acid (4TMS)	225.78			Raffinose (11TMS)	117.27
Glucopyranose, D- (5TMS)	356.96				
Glycerophosphoglycerol (5TMS)	407.22				
Fructose-6-phosphate (1MEOX) (6TMS) MP	837.52				

Mannose-6-phosphate (1MEOX) (6TMS) MP	781.53
Glucose-6-phosphate (1MEOX) (6TMS) BP	601.68
Sucrose (8TMS)	130.80
Galactinol (9TMS)	250.40
Raffinose (11TMS)	568.71

Arabidopsis and Anastatica

**Species significant metabolites**	**F-ratio**	**Treatment (salt) significant metabolites**	**F-ratio**	**Species x Treatment significant metabolites**	**F-ratio**
Valine (1TMS)	334.28	Valine (1TMS)	9.60	Alanine (2TMS)	6.96
Alanine (2TMS)	36.14	Glycine (2TMS)	140.19	Glycine (2TMS)	8.09
Proline (1TMS)	17.20	Proline (1TMS)	376.64	Valine (2TMS)	78.61
Valine (2TMS)	57.12	Valine (2TMS)	36.77	Benzoic acid, (1TMS)	13.09
Benzoic acid, (1TMS)	48.53	Benzoic acid, (1TMS)	5.34	Serine (2TMS)	27.31
Serine (2TMS)	8.84	Serine (2TMS)	28.30	Phosphoric acid (3TMS)	31.17
Glycerol (3TMS)	119.55	Glycerol (3TMS)	10.76	Proline (2TMS)	23.26
Phosphoric acid (3TMS)	271.19	Phosphoric acid (3TMS)	5.77	Glycine (3TMS)	39.46
Glycine (3TMS)	67.50	Proline (2TMS)	122.93	Succinic acid (2TMS)	45.24
Succinic acid (2TMS)	144.56	Glycine (3TMS)	11.99	Glyceric acid (3TMS)	13.31
Glyceric acid (3TMS)	86.79	Succinic acid (2TMS)	7.81	Uracil (2TMS)	49.14
Uracil (2TMS)	72.98	Glyceric acid (3TMS)	8.19	Fumaric acid (2TMS)	11.69
Fumaric acid (2TMS)	1383.03	Uracil (2TMS)	39.55	Serine (3TMS)	20.54
Threonine (3TMS)	31.34	Serine (3TMS)	24.35	Alanine (3TMS)	9.45
Aspartic acid (2TMS)	361.82	Alanine (3TMS)	3.90	Threonine (3TMS)	15.69
Malic acid (3TMS)	287.98	Threonine (3TMS)	103.17	Aspartic acid (2TMS)	81.21
Pyroglutamic acid (2TMS)	180.09	Aspartic acid (2TMS)	40.07	Malic acid (3TMS)	20.05
Butanoic acid, 4-amino- (3TMS)	48.70	Malic acid (3TMS)	3.86	Threitol (4TMS)	6.62
Serine (4TMS)	127.56	Pyroglutamic acid (2TMS)	13.03	Pyroglutamic acid (2TMS)	27.23
Proline [+CO2] (2TMS)	620.36	Butanoic acid, 4-amino- (3TMS)	83.33	Butanoic acid, 4-amino- (3TMS)	33.59
Xylose (1MEOX) (4TMS) MP	73.01	Erythronic acid (4TMS)	15.18	Erythronic acid (4TMS)	5.48
Lyxose (1MEOX) (4TMS) MP	255.44	Serine (4TMS)	20.94	Serine (4TMS)	114.18
Glucose, 1,6-anhydro, beta- (3TMS)	108.97	Proline [+CO2] (2TMS)	187.78	Proline [+CO2] (2TMS)	9.30
Glucopyranose [-H20] (4TMS)	11.36	Xylose (1MEOX) (4TMS) MP	3.44	Lyxose (1MEOX) (4TMS) MP	3.51
Citric acid (4TMS)	113.60	Arabinose (1MEOX) (4TMS) BP	16.07	Arabinose (1MEOX) (4TMS) BP	3.75
Psicose (1MEOX) (5TMS) BP	113.27	Glucose, 1,6-anhydro, beta- (3TMS)	16.56	Glucose, 1,6-anhydro, beta- (3TMS)	4.33
Psicose (1MEOX) (5TMS) MP	189.96	Glycerol-3-phosphate (4TMS)	12.83	Glycerol-3-phosphate (4TMS)	23.27

Glucose (1MEOX) (5TMS) MP	99.55	Citric acid (4TMS)	46.50	Shikimic acid (4TMS)	4.87
Galactose (1MEOX) (5TMS) BP	218.20	Psicose (1MEOX) (5TMS) BP	10.32	Citric acid (4TMS)	24.00
Ascorbic acid (4TMS)	75.01	Psicose (1MEOX) (5TMS) MP	17.62	Psicose (1MEOX) (5TMS) BP	4.46
Glucopyranose, D- (5TMS)	97.52	Glucose (1MEOX) (5TMS) MP	10.88	Psicose (1MEOX) (5TMS) MP	3.59
Inositol, myo- (6TMS)	35.43	Galactose (1MEOX) (5TMS) BP	48.94	Glucose (1MEOX) (5TMS) MP	11.44
Glycerophosphoglycerol (5TMS)	57.91	Ascorbic acid (4TMS)	3.54	Galactose (1MEOX) (5TMS) BP	11.25
Fructose-6-phosphate (1MEOX) (6TMS) MP	191.84	Inositol, myo- (6TMS)	95.07	Glucopyranose, D- (5TMS)	4.43
Mannose-6-phosphate (1MEOX) (6TMS) MP	475.22	Glycerophosphoglycerol (5TMS)	11.85	Inositol, myo- (6TMS)	27.09
Glucose-6-phosphate (1MEOX) (6TMS) BP	118.66	Fructose-6-phosphate (1MEOX) (6TMS) MP	7.97	Glycerophosphoglycerol (5TMS)	39.19
Sucrose (8TMS)	8.11	Mannose-6-phosphate (1MEOX) (6TMS) MP	17.99	Fructose-6-phosphate (1MEOX) (6TMS) MP	26.96
Galactinol (9TMS)	38.82	Glucose-6-phosphate (1MEOX) (6TMS) BP	9.23	Mannose-6-phosphate (1MEOX) (6TMS) MP	43.09
Raffinose (11TMS)	1647.06	Sucrose (8TMS)	24.79	Glucose-6-phosphate (1MEOX) (6TMS) BP	12.13
		Galactinol (9TMS)	6.01	Sucrose (8TMS)	11.48
		Raffinose (11TMS)	192.48	Galactinol (9TMS)	6.22
				Raffinose (11TMS)	12.40

Eutrema and Anastatica

**Species significant metabolites**	**F-ratio**	**Treatment (salt) significant metabolites**	**F-ratio**	**Species x Treatment significant metabolites**	**F-ratio**
Valine (1TMS)	495.15	Valine (1TMS)	21.66	Alanine (2TMS)	5.38
Glycine (2TMS)	403.05	Alanine (2TMS)	4.60	Glycine (2TMS)	5.35
Butyro-1,4-lactam (1TMS)	11.27	Glycine (2TMS)	353.55	Proline (1TMS)	20.02
Proline (1TMS)	98.05	Proline (1TMS)	676.83	Valine (2TMS)	188.40
Glycerol (3TMS)	9.15	Valine (2TMS)	294.03	Serine (2TMS)	78.46
Phosphoric acid (3TMS)	18.67	Serine (2TMS)	6.19	Proline (2TMS)	103.22
Proline (2TMS)	44.45	Glycerol (3TMS)	5.53	Glycine (3TMS)	14.57
Glycine (3TMS)	238.92	Phosphoric acid (3TMS)	18.74	Succinic acid (2TMS)	17.56
Succinic acid (2TMS)	75.82	Proline (2TMS)	28.26	Glyceric acid (3TMS)	48.63
Glyceric acid (3TMS)	53.85	Glycine (3TMS)	4.24	Fumaric acid (2TMS)	14.20
Uracil (2TMS)	4.93	Succinic acid (2TMS)	81.51	Serine (3TMS)	29.44
Fumaric acid (2TMS)	325.41	Glyceric acid (3TMS)	20.93	Threonine (3TMS)	53.08
Serine (3TMS)	81.04	Uracil (2TMS)	9.31	Methionine (1TMS)	22.17
Alanine (3TMS)	14.84	Fumaric acid (2TMS)	8.41	Aspartic acid (2TMS)	8.54
Threonic acid-1,4-lactone (2TMS)	69.38	Serine (3TMS)	36.93	Glutamine [-H2O] (2TMS) BP	4.77
Threonine (3TMS)	12.27	Alanine (3TMS)	11.39	Malic acid (3TMS)	34.64
Methionine (1TMS)	42.47	Threonic acid-1,4-lactone	7.96	Threitol (4TMS)	4.40

		(2TMS)			
Aspartic acid (2TMS)	44.73	Threonine (3TMS)	88.32	Pyroglutamic acid (2TMS)	3.89
Glutamine [-H2O] (2TMS) BP	11.68	Malic acid (3TMS)	84.45	Butanoic acid, 4-amino- (3TMS)	39.16
Malic acid (3TMS)	211.06	Threitol (4TMS)	4.15	Glutamic acid (2TMS)	6.77
Threitol (4TMS)	48.52	Pyroglutamic acid (2TMS)	11.24	Erythronic acid (4TMS)	8.92
Pyroglutamic acid (2TMS)	209.92	Butanoic acid, 4-amino- (3TMS)	5.86	Threonic acid (4TMS)	30.89
Erythronic acid (4TMS)	145.82	Erythronic acid (4TMS)	41.13	Serine (4TMS)	65.13
Threonic acid (4TMS)	297.30	Threonic acid (4TMS)	67.21	Proline [+CO2] (2TMS)	28.24
Serine (4TMS)	196.03	Serine (4TMS)	64.08	Glutamic acid (3TMS)	11.96
Proline [+CO2] (2TMS)	25.10	Proline [+CO2] (2TMS)	552.29	Phenylalanine (2TMS)	6.06
Glutamic acid (3TMS)	34.29	Arabinose (1MEOX) (4TMS) BP	15.27	Arabinose (1MEOX) (4TMS) BP	6.61
Xylose (1MEOX) (4TMS) MP	158.92	Putrescine (4TMS)	102.01	Glucose, 1,6-anhydro, beta- (3TMS)	12.84
Lyxose (1MEOX) (4TMS) MP	177.45	Glycerol-3-phosphate (4TMS)	64.47	Putrescine (4TMS)	189.59
Arabinose (1MEOX) (4TMS) BP	74.19	Ribonic acid (5TMS)	18.23	Glycerol-3-phosphate (4TMS)	24.91
Glucose, 1,6-anhydro, beta- (3TMS)	38.46	Shikimic acid (4TMS)	13.60	Ribonic acid (5TMS)	49.84
Putrescine (4TMS)	744.69	Citric acid (4TMS)	179.18	Citric acid (4TMS)	77.39
Glycerol-3-phosphate (4TMS)	25.58	Psicose (1MEOX) (5TMS) BP	4.82	Psicose (1MEOX) (5TMS) MP	3.96
Ribonic acid (5TMS)	193.78	Psicose (1MEOX) (5TMS) MP	9.86	Galactose (1MEOX) (5TMS) BP	10.57
Glucopyranose [-H20] (4TMS)	22.81	Ascorbic acid (4TMS)	13.19	Ascorbic acid (4TMS)	4.52
Citric acid (4TMS)	70.16	Glucopyranose, D- (5TMS)	15.43	Glucopyranose, D- (5TMS)	5.40
Psicose (1MEOX) (5TMS) BP	398.97	Inositol, myo- (6TMS)	265.65	Inositol, myo- (6TMS)	22.11
Dehydroascorbic acid dimer (2MEOX) MP	58.99	Glycerophosphoglycerol (5TMS)	68.38	Glycerophosphoglycerol (5TMS)	4.98
Psicose (1MEOX) (5TMS) MP	610.59	Mannose-6-phosphate (1MEOX) (6TMS) MP	6.49	Fructose-6-phosphate (1MEOX) (6TMS) MP	12.45
Glucose (1MEOX) (5TMS) MP	14.94	Sucrose (8TMS)	53.59	Mannose-6-phosphate (1MEOX) (6TMS) MP	5.84
Galactose (1MEOX) (5TMS) BP	71.12	Raffinose (11TMS)	19.83	Sucrose (8TMS)	24.53
Ascorbic acid (4TMS)	56.16			Raffinose (11TMS)	41.78
Glucopyranose, D- (5TMS)	12.42				
Inositol, myo- (6TMS)	43.94				
Glycerophosphoglycerol (5TMS)	261.07				
Fructose-6-phosphate (1MEOX) (6TMS) MP	325.33				
Mannose-6-phosphate (1MEOX) (6TMS) MP	225.74				
Glucose-6-phosphate (1MEOX) (6TMS) BP	149.45				

Sucrose (8TMS)	152.83
Galactinol (9TMS)	86.63
Raffinose (11TMS)	70.10

Supplementary Table S3. Ono-way ANOVA (*P* < 0.05) of metabolite response of soil-grown plants (Exp. 1).

***Arabidopsis*_significant**	**F-ratio**	***E. salsugineum*_significant**	**F-ratio**	***A. hierochuntica*_significant**	**F-ratio**
Valine (2TMS)	7.66	Urea (2TMS)	175.48	Valine (2TMS)	8.40
Urea (2TMS)	8.83	Serine (2TMS)	11.35	Serine (2TMS)	12.33
Serine (2TMS)	92.81	Phosphoric acid (3TMS)	4.28	Phosphoric acid (3TMS)	7.11
Phosphoric acid (3TMS)	15.03	Glycine (3TMS)	55.54	Succinic acid (2TMS)	4.63
Glycine (3TMS)	10.10	Succinic acid (2TMS)	6.06	Alanine (3TMS)	24.15
Succinic acid (2TMS)	20.81	Glyceric acid (3TMS)	16.64	Threonine, allo- (3TMS)	15.17
Glyceric acid (3TMS)	4.94	Aspartic acid (2TMS)	55.26	Aspartic acid (2TMS)	18.09
Fumaric acid (2TMS)	16.64	Glutamine [-H2O] (2TMS) BP	207.08	Pyroglutamic acid (2TMS)	7.48
Alanine (3TMS)	36.69	Malic acid (3TMS)	22.78	Threonic acid (4TMS)	8.87
Threonine, allo- (3TMS)	20.07	Glutamic acid (2TMS)	15.98	Proline (pooled)	163.01
Glutamine [-H2O] (2TMS) BP	9.86	Threonic acid (4TMS)	9.16	Ribonic acid (5TMS)	3.96
Malic acid (3TMS)	10.17	Proline (pooled)	50.15	Citric acid (4TMS)	29.69
Threonic acid (4TMS)	6.39	Ribonic acid (5TMS)	8.83	Fructose (1MEOX) (5TMS) BP	12.94
Proline (pooled)	83.42	Citric acid (4TMS)	28.51	Glucose (1MEOX) (5TMS) MP	10.86
Glucopyranose [-H20] (4TMS)	10.53	Fructose (1MEOX) (5TMS) BP	4.56	Inositol, myo- (6TMS)	29.33
Fructose (1MEOX) (5TMS) BP	4.39	Inositol, myo- (6TMS)	24.04	Fructose-6-phosphate (1MEOX) (6TMS) MP	32.56
Ascorbic acid (4TMS)	6.63	Fructose-6-phosphate (1MEOX) (6TMS) MP	14.10	Galactinol (9TMS)	20.71
Inositol, myo- (6TMS)	10.26	Glucose-6-phosphate (1MEOX) (6TMS) BP	11.44	Raffinose (11TMS)	14.21
Sucrose (8TMS)	13.85	Sucrose (8TMS)	6.25		
Galactinol (9TMS)	32.85	Galactinol (9TMS)	14.19		
Raffinose (11TMS)	59.79	Raffinose (11TMS)	258.45		

Supplementary Table S3. Ono-way ANOVA (*P* < 0.05) of metabolite response of soil-grown plants (Exp. 2).

***Arabidopsis*_significant**	**F-ratio**	***E. salsugineum*_significant**	**F-ratio**	***A. hierochuntica*_significant**	**F-ratio**
Valine (1TMS)	4.44	Valine (1TMS)	43.10	Valine (1TMS)	6.35
Glycine (2TMS)	105.74	Glycine (2TMS)	304.20	Alanine (2TMS)	6.91
Proline (1TMS)	114.16	Proline (1TMS)	154.57	Glycine (2TMS)	44.23
Benzoic acid, (1TMS)	34.45	Malonic acid (2TMS)	67.06	Proline (1TMS)	382.76
Glycerol (3TMS)	4.47	Valine (2TMS)	109.81	Valine (2TMS)	243.62
Phosphoric acid (3TMS)	30.40	Serine (2TMS)	26.53	Serine (2TMS)	42.33
Proline (2TMS)	32.19	Phosphoric acid (3TMS)	6.04	Glycerol (3TMS)	6.32
Glycine (3TMS)	66.07	Proline (2TMS)	8.84	Phosphoric acid (3TMS)	6.11
Succinic acid (2TMS)	37.66	Glycine (3TMS)	32.79	Proline (2TMS)	143.45
Glyceric acid (3TMS)	22.05	Succinic acid (2TMS)	55.49	Glycine (3TMS)	9.59
Uracil (2TMS)	63.79	Glyceric acid (3TMS)	195.21	Succinic acid (2TMS)	10.52
Fumaric acid (2TMS)	4.21	Uracil (2TMS)	8.13	Glyceric acid (3TMS)	7.57
Threonine (3TMS)	28.88	Fumaric acid (2TMS)	8.27	Fumaric acid (2TMS)	10.81
Aspartic acid (2TMS)	116.16	Serine (3TMS)	11.04	Serine (3TMS)	34.95
Malic acid (3TMS)	7.83	Threonic acid-1,4-lactone (2TMS)	16.20	Alanine (3TMS)	11.19
Pyroglutamic acid (2TMS)	30.28	Methionine (1TMS)	19.57	Threonine (3TMS)	100.86
Butanoic acid, 4-amino- (3TMS)	54.52	Aspartic acid (2TMS)	5.56	Methionine (1TMS)	11.00
Serine (4TMS)	24.06	Glutamine [-H2O] (2TMS) BP	21.67	Aspartic acid (2TMS)	4.45
Proline [+CO2] (2TMS)	88.87	Malic acid (3TMS)	76.98	Malic acid (3TMS)	15.57
Arabinose (1MEOX) (4TMS) BP	5.14	Erythronic acid (4TMS)	51.32	Threitol (4TMS)	4.92
Glucose, 1,6-anhydro, beta- (3TMS)	32.17	Threonic acid (4TMS)	165.26	Pyroglutamic acid (2TMS)	4.86
Shikimic acid (4TMS)	4.17	Proline [+CO2] (2TMS)	251.19	Butanoic acid, 4-amino- (3TMS)	86.83
Citric acid (4TMS)	43.15	Glutamic acid (3TMS)	13.58	Glutamic acid (2TMS)	15.39
Fructose (1MEOX) (5TMS) BP	15.46	Glucose, 1,6-anhydro, beta- (3TMS)	6.12	Erythronic acid (4TMS)	12.47
Fructose (1MEOX) (5TMS) MP	19.35	Putrescine (4TMS)	44.42	Threonic acid (4TMS)	18.73
Glucose (1MEOX) (5TMS) MP	70.40	Glycerol-3-phosphate (4TMS)	8.33	Serine (4TMS)	161.55
Galactose (1MEOX) (5TMS) BP	136.00	Ribonic acid (5TMS)	57.14	Proline [+CO2] (2TMS)	109.22
Ascorbic acid (4TMS)	4.45	Shikimic acid (4TMS)	10.18	Arabinose (1MEOX) (4TMS) BP	24.03
Glucopyranose, D- (5TMS)	14.15	Citric acid (4TMS)	474.07	Glucose, 1,6-anhydro, beta- (3TMS)	7.03
Hexadecanoic acid (1TMS)	4.09	Ascorbic acid (4TMS)	12.65	Putrescine (4TMS)	45.67
Inositol, myo- (6TMS)	27.38	Glucopyranose, D- (5TMS)	31.66	Glycerol-3-phosphate (4TMS)	52.75
Glycerophosphoglycerol (5TMS)	10.99	Inositol, myo- (6TMS)	105.64	Ribonic acid (5TMS)	5.96

Fructose-6-phosphate (1MEOX) (6TMS) MP	22.19	Glycerophosphoglycerol (5TMS)	15.38	Citric acid (4TMS)	28.53
Mannose-6-phosphate (1MEOX) (6TMS) MP	39.53	Fructose-6-phosphate (1MEOX) (6TMS) MP	8.24	Fructose (1MEOX) (5TMS) BP	4.45
Glucose-6-phosphate (1MEOX) (6TMS) BP	18.10	Sucrose (8TMS)	18.28	Fructose (1MEOX) (5TMS) MP	7.20
Galactinol (9TMS)	9.78			Galactose (1MEOX) (5TMS) BP	5.77
Raffinose (11TMS)	200.50			Inositol, myo- (6TMS)	69.06
				Glycerophosphoglycerol (5TMS)	59.08
				Fructose-6-phosphate (1MEOX) (6TMS) MP	8.54
				Mannose-6-phosphate (1MEOX) (6TMS) MP	13.19
				Sucrose (8TMS)	33.41
				Raffinose (11TMS)	43.90
